# Longitudinal analysis of biomarker data from a personalized nutrition platform in healthy subjects

**DOI:** 10.1038/s41598-018-33008-7

**Published:** 2018-10-02

**Authors:** Kenneth Westerman, Ashley Reaver, Catherine Roy, Margaret Ploch, Erin Sharoni, Bartek Nogal, David A. Sinclair, David L. Katz, Jeffrey B. Blumberg, Gil Blander

**Affiliations:** 1 InsideTracker, Cambridge, Massachusetts United States of America; 2 The Friedman School of Nutrition Science and Policy at Tufts University, Boston, Massachusetts United States of America; 3 Yale University Prevention Research Center, Griffin Hospital, Yale University School of Medicine, Derby, Connecticut United States of America; 4 Department of Genetics, Harvard Medical School, Boston, Massachusetts United States of America; 5 Department of Pharmacology, The University of New South Wales, Sydney, New South Wales Australia

## Abstract

The trend toward personalized approaches to health and medicine has resulted in a need to collect high-dimensional datasets on individuals from a wide variety of populations, in order to generate customized intervention strategies. However, it is not always clear whether insights derived from studies in patient populations or in controlled trial settings are transferable to individuals in the general population. To address this issue, a longitudinal analysis was conducted on blood biomarker data from 1032 generally healthy individuals who used an automated, web-based personalized nutrition and lifestyle platform. The study had two main aims: to analyze correlations between biomarkers for biological insights, and to characterize the effectiveness of the platform in improving biomarker levels. First, a biomarker correlation network was constructed to generate biological hypotheses that are relevant to researchers and, potentially, to users of personalized wellness tools. The correlation network revealed expected patterns, such as the established relationships between blood lipid levels, as well as novel insights, such as a connection between neutrophil and triglyceride concentrations that has been suggested as a relevant indicator of cardiovascular risk. Next, biomarker changes during platform use were assessed, showing a trend toward normalcy for most biomarkers in those participants whose values were out of the clinically normal range at baseline. Finally, associations were found between the selection of specific interventions and corresponding biomarker changes, suggesting directions for future study.

## Introduction

Personalized nutrition approaches have the potential to improve the effectiveness of lifestyle-based disease management and prevention, being both more efficacious and more motivating to the individual^1^. Currently, the personalized nutrition research landscape is focused on the integration of genetic data^2,3^. However, recommendations for nutritional interventions based solely on genetic data are limited, as they reflect only an individual’s genetic predisposition and fail to incorporate relevant modifiable risk factors within the individual’s surrounding environment^4,5^. Serum biomarkers, on the other hand, are well-suited for personalized nutrition assessment and monitoring as they provide real-time snapshots – reflections of an individual’s current metabolic or physiological state. Importantly, the information provided by serum biomarkers is easily trackable and readily actionable, as these markers change over time in response to nutrition, exercise, and other lifestyle factors.

The shift toward personalized health and medicine requires a parallel shift in measurement paradigms. To achieve truly personalized, efficacious recommendations, measurement must become more comprehensive and offer complementary views of biological processes. With regard to personalized health, this can be accomplished in two ways: (1) with repeated or longitudinal measurements, and (2) with the measurement of a broader range of biomarkers. The movement towards systems approaches to healthcare has been termed “P4” medicine (predictive, personalized, preventive, and participatory), and it is partially defined by this type of comprehensive measurement^6^. Several recent proof-of-concept studies have shown the power of the P4 approach by measuring and analyzing very large sets of biomarkers, including “omics” data, to better understand transitions between health and disease states and discover new predictive biomarkers^7,8^. However, the scale of these approaches is limited by costly and labor-intensive requirements for data collection, interpretation, and communication with the participating individual(s). To that end, employing algorithmic automation can help make these interventions and comprehensive data measurements more scalable.

Combining the power of automation with the concepts of biological and behavioral personalization, we developed an online platform to produce individually customized nutrition and lifestyle recommendations for healthy adults, based on a panel of serum biomarkers. The scalable nature of the platform has allowed us to generate longitudinal biomarker data on 1032 generally healthy adults. We sought to investigate the resulting dataset to (1) find biological insights and generate hypotheses for future research, particularly those that may be more relevant to generally healthy individuals, and (2) assess longitudinal changes in biomarker levels in platform participants over time. We describe a series of analyses of this dataset, discovering expected and novel correlations between biomarker changes, trends toward normalcy in biomarker levels during use of the platform, and associations between specific intervention choices and biomarker changes.

## Results

### Study platform and population

**Figure 1:**
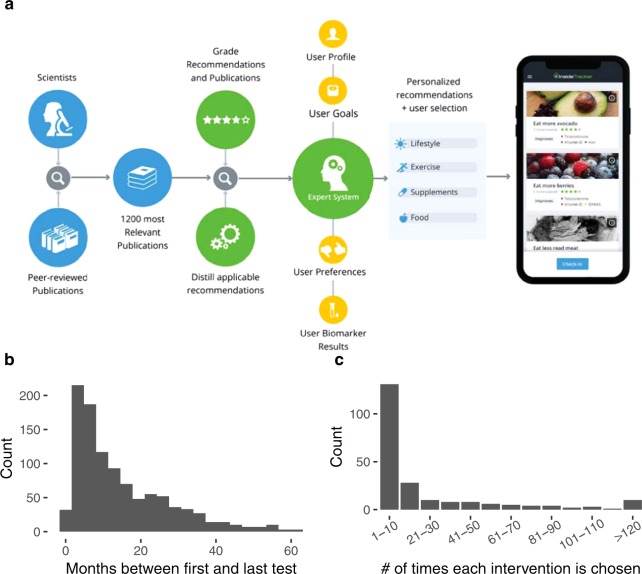
The InsideTracker platform provides a means to generate longitudinal biomarker measurements before and after delivery of a set of personalized nutrition and health recommendations. (**a**) Graphical description of the InsideTracker algorithm and platform. (**b**) Histogram of time between tests. (**c**) Histogram of intervention choice frequencies.

The full population explored here consisted of apparently healthy individuals who used a web-based personalized nutrition and lifestyle platform called InsideTracker (see Methods section for details). Participants included in this analysis received at least two blood tests along with the associated personalized nutrition and lifestyle recommendations. This group consisted of 1032 individuals in total across a broad age range (Table 1). Participants were recommended to follow interventions for a minimum of 3 months and then retest, however, follow-up testing dates ranged from less than one month post-baseline to 60 months post-baseline (Fig. 1).

**Table 1:** Population demographics.

	Male	Female
# of participants	672	360
Age (years)	43 (16)	40 (16)
	85% White	84% White
	6% Asian	10% Asian
Ethnicity	4% Black	3% Black
	3% Hispanic	3% Hispanic
	2% Indian	0% Indian
BMI (kg/m2)	25.2 (4.3)	22.3 (4.3)
Exercise (hrs/wk)	4.0 (4.8)	4.2 (4.0)

Note: Numeric values are presented as: median (IQR).

The recommendations given by the platform stem from a system that synthesizes a broad scientific literature base for interventions associated with changes in biomarker levels. Each individual was presented with a variety of food, supplement, and lifestyle recommendations, based on their baseline biomarker levels, from which they selected a subset to follow. Thus, we looked at the extent to which participants tended to choose truly unique or personalized sets of interventions (Fig. 1c). With the exception of a small set of commonly-chosen interventions (e.g. oatmeal consumption; list of most common interventions in Supplementary Table S1), the bulk of the selections consisted of those that tended to be chosen relatively infrequently.

### Longitudinal biomarker analysis reveals novel biological correlations

**Figure 2:**
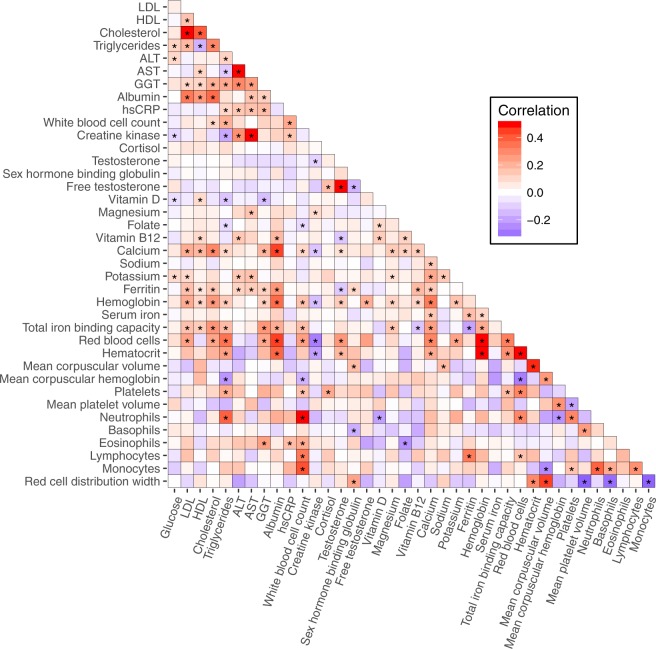
Heatmap of the overall correlation matrix. Colors correspond to the magnitude of Spearman correlations between changes in each pair of biomarkers. Asterisks indicate multiple test-corrected p < 0.05.

We explored the set of longitudinal biomarker data by calculating the correlations between changes in biomarkers (i.e. a positive correlation indicates that changes in one biomarker tend to be in the same direction as those of its partner). Figure 2 illustrates the strength of Spearman correlations between changes in each pair of biomarkers. We identified well-established and physiologically expected correlations (e.g. alanine aminotransferase and aspartate aminotransferase) as well as novel and less-explored relationships (see Discussion section for more details).

**Figure 3:**
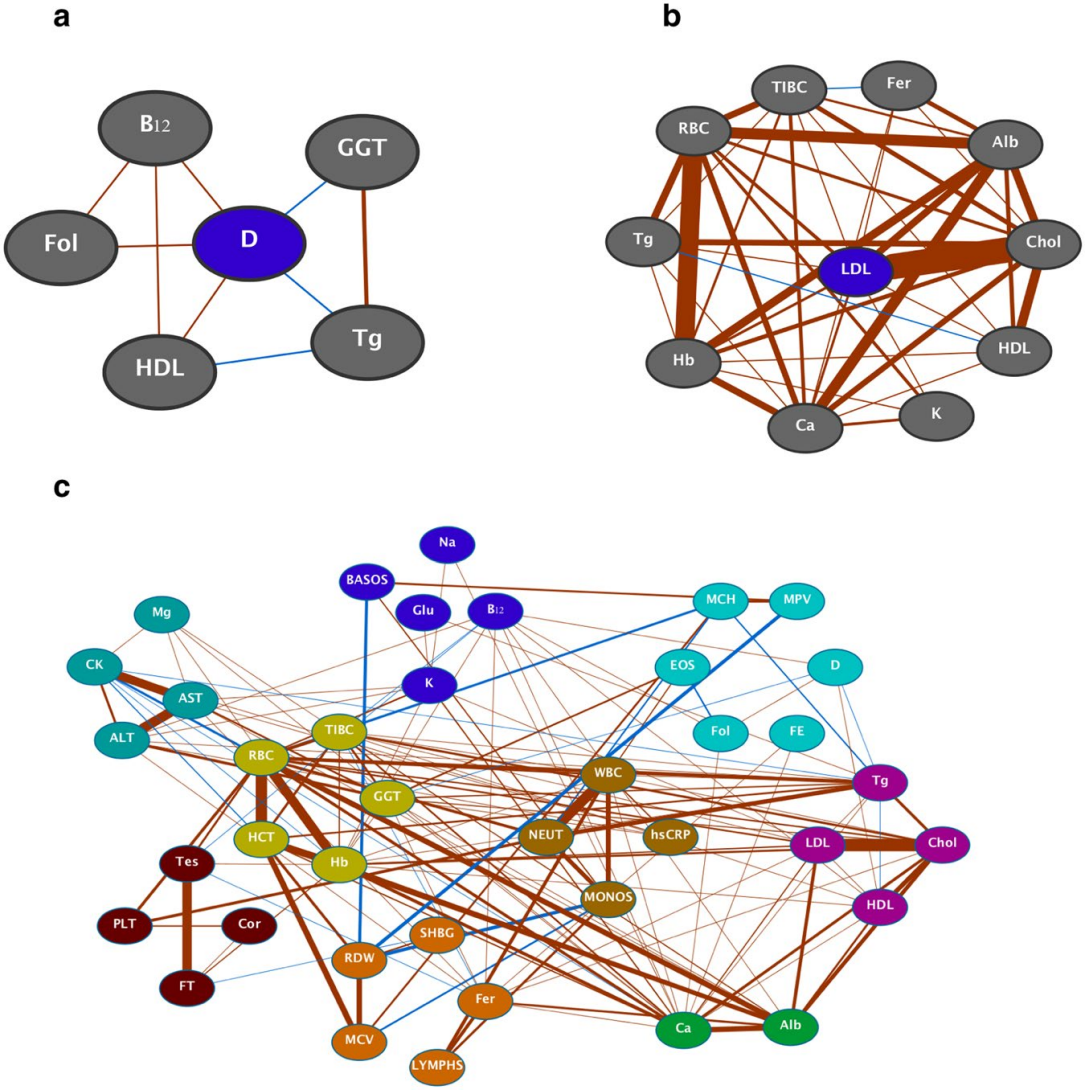
Further investigation of the longitudinal correlation network. Connections are displayed in network format, with edges corresponding to Spearman correlations with BH-corrected p < 0.05. Edge weights are proportional to the correlation strength, and colors correspond to the direction of association (red is positive, blue is negative). (**a**,**b**) Sub-networks consisting of only nodes connected to vitamin D and LDL, respectively. (**c**) Hierarchical clustering-based community detection results. Edges are as above, with biomarker nodes colored according to their identified cluster. See Supplementary Table S2 for full list of abbreviations.

In order to generate further insights from this analysis, we looked more deeply at the “neighborhoods” of specific biomarkers, displayed here in a network format (Fig. 3a,b). Edge weights correspond to the Spearman correlation strengths, and are only shown if statistically significant at p < 0.05 after correction for multiple comparisons. We focused here on vitamin D and LDL, as they have important implications for a spectrum of health-related physiological processes, and are commonly sub-optimal even in otherwise healthy individuals^9,10^. Vitamin D revealed links with biomarkers representing a range of biological processes, including nutrient intake, liver function, and lipid metabolism. LDL correlated with markers related to iron storage, lipid metabolism, and electrolyte status.

As a further method of probing the correlation structure, we performed hierarchical clustering on the set of biomarkers, using a basic distance metric derived from the full pairwise Spearman correlation matrix. There was no easily identifiable cut point based on visual inspection of the dendrogram, so a dynamic tree cutting algorithm^11^ was used to find an optimal set of sub-modules (depicted in network format in Fig. 3c). Some of these modules illustrated anticipated relationships, such as a cluster including hsCRP and white blood cells as well as another linking classical lipid profile components. However, less intuitive findings also arose, including the clustering of platelets with hormone status and magnesium concentration with biomarkers of muscle and liver stress.

Two potential complicating factors for the relationships discovered here are very long breaks between tests as well as sex. We performed sensitivity analyses to understand whether these factors notably affected our conclusions by re-calculating the same longitudinal correlations in stratified datasets. In a group consisting of only individuals who re-tested within 2 years, the Pearson correlation between correlation coefficients (time-filtered subset vs. entire population) was found to be 0.987. After stratifying by sex, these equivalent correlations were found to be 0.961 for males and 0.878 for females. In addition to these broad similarities, we confirmed that specific hypotheses (correlations between biomarker changes) were also largely consistent (see Supplementary Table S3).

### Platform use associates with improvements in biomarker levels in out-of-range participants

Next, we investigated the changes that occurred from baseline to follow-up in each biomarker separately. Results in our entire study population can be found in Supplementary Table S4. As these results are complicated by the difficulty of defining “improvement” in individuals with normal biomarker values at baseline, we focused on those individuals whose baseline values were outside of the clinically acceptable range for a given marker. It is more clear in these participants which direction of change constitutes improvement; further, these individuals would have received recommendations from the platform targeting that biomarker. A single “direction of risk” was defined for each biomarker as the direction in which individuals are most commonly found out-of-range (e.g. for blood glucose, an upper rather than lower limit was set). Participants tended to be out-of-range for only a few biomarkers each (full distribution in Fig. S1), with 906 individuals out-of-range for at least one biomarker. We analyzed the set of 17 biomarkers for which at least 20 participants were out-of-range at baseline, and observed substantial changes from baseline to follow-up in almost all markers examined (Table 2). As there was no randomization involved in this analysis, we do not infer any causality of platform use or identify which of its components may have driven the observed changes. Nonetheless, these observations suggest a potential influence of the platform on biomarkers in out-of-range individuals.

**Table 2:** Change in biomarker levels for participants out-of-range at baseline.

Biomarker	Baseline median (IQR)	Follow-up median (IQR)	P-value	Sample size	Out-of-range threshold
Vitamin D^a^	23.7 (6.05)	32.4 (15)	<0.001	383	<30
LDL	149 (27)	139 (41.5)	<0.001	303	>130
Creatine kinase	353 (213.5)	241 (253)	<0.001	227	>230
Glucose	105 (7)	97 (14)	<0.001	77	>100
HDL	42.9 (8)	45 (11.5)	<0.001	215	<50
Cholesterol	221 (33)	217 (47)	<0.001	349	>200
ALT	43 (20.5)	30 (18.5)	<0.001	59	>46(M); >29(F)
Triglycerides	191 (58.75)	144.5 (108.8)	<0.001	88	>150
Cortisol	24.3 (3.7)	19.9 (9)	<0.001	51	>22
Ferritin	8 (4.5)	20.5 (22.5)	<0.001	30	<20(M); <10(F)
hsCRP	4.9 (3.16)	2.5 (4.4)	<0.001	55	>3
AST	51 (13)	30 (12)	<0.001	29	>40
Testosterone^b^	219 (62)	406 (337)	<0.001	25	<250(M); <0(F)
Eosinophils	267.5 (103.5)	234 (131.8)	<0.001	40	>200
Mean corpuscular hemoglobin concentration	31.1 (0.95)	32 (2.8)	0.066	36	<32
Sex hormone binding globulin	64 (38)	64 (34)	0.132	325	>40
Free testosterone^b^	9.9 (4.4)	10 (4)	0.161	296	<46(M); <0(F)

^a^P-values for vitamin D were calculated after an adjustment of 2.5 mg/dL down for tests taken during the summer (see Methods).

^b^Analyses were stratified by sex for testosterone and free testosterone, but an insufficient amount of females were out-of-range for either marker in our dataset. Results shown for these two markers are based on males only.

Changes in weight/body mass index are an important potential explanatory factor of the observed biomarker level changes over time. Due to weight data being collected independently of biomarker data, follow-up weights were available only for 428 participants and were not always concurrent with the follow-up blood tests, so were not analyzed formally with the blood biomarkers. However, for the participants with follow-up weights available, we observed a small decrease in mean BMI (mean change of −0.22, p = 0.043 from paired t-test).

### Intervention choice data suggests biomarker-specific intervention effectiveness

**Figure 4:**
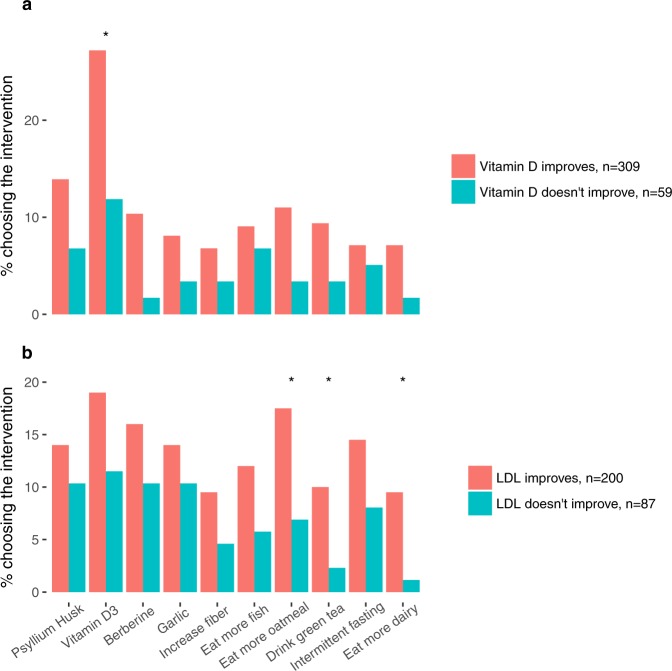
Associations between intervention choice and biomarker changes. All individuals out-of-range for vitamin D (**a**) and LDL (**b**) at baseline are stratified based on the presence or absence of improvement from baseline to follow-up test. The y-axis corresponds to the percentage of each group (improved/not improved) having chosen the intervention. The x-axis corresponds to the 10 most commonly chosen interventions across all participants. Asterisks indicate nominal significance (uncorrected p < 0.05, Chi-square test).

We sought to find any patterns that may exist between participants’ choice of specific lifestyle and nutrition interventions and the observed changes in any out-of-range biomarkers. Because such an analysis requires notable numbers of participants to both be out-of-range at baseline and choose a specific intervention, we had potentially low power to detect these relationships. So, we chose to focus on vitamin D and LDL, which are commonly out-of-range, and the top ten most commonly chosen interventions across all participants (four of which are dietary supplements) (Fig. 4). For each combination of biomarker and intervention, we stratified the set of out-of-range participants into those who improved over time and those who did not, and subsequently compared the fractions of participants having chosen the intervention between groups. Though we lack rigorous measures of adherence, this approach may provide preliminary clues as to which interventions are more effective in affecting change in specific biomarkers.

We observed an overall bias toward improvement in individuals who chose interventions, as seen in the consistently greater improvement proportion across interventions in both vitamin D and LDL. For vitamin D, one specific association (vitamin D supplementation) reached nominal significance, suggesting that there was reasonable adherence at least to this particular intervention. For LDL, nominal associations were found with increased consumption of oatmeal, green tea, and dairy. None of the associations for any biomarker retained statistical significance after Benjamini-Hochberg correction for multiple testing, possibly due to the low effective sample size for this analysis.

## Discussion

We describe an analysis of the InsideTracker dataset consisting of longitudinal blood biomarker measurements in a generally healthy population. These data straddle the exposure of the participants to a series of nutrition and lifestyle recommendations based on baseline dietary behavior, preferences, and health markers. The automated and scalable nature of the study platform allowed the generation of data for a relatively large number of participants, which in turn enabled the systems-level biomarker analysis.

We took advantage of the longitudinal nature of our data to explore correlations in changes over time between biomarkers. There is a precedent for the notion that some biological patterns are revealed only upon changes to the system rather than at baseline–this has been explored well in the context of acute challenges to metabolism^12,13^ but, in contrast, the study platform recommendations act as a longer-term perturbation. After calculating pairwise correlations between changes in biomarkers, we observed both expected and novel connections.

The observations in this dataset with respect to vitamin D were consistent with existing literature. The relationship between vitamin D and a range of health parameters has been extensively explored in recent years. Although observational studies support an association between higher vitamin D levels and a more favorable lipid profile, intervention trials have generally failed to reach the same conclusion^14,15^. Our results support the observational associations, and include a longitudinal component–these associations exist not only in cross-sectional analyses, but also in medium-term (months to a few years) changes. Though some or all of this effect may be due to lifestyle changes rather than a causal role of vitamin D, our results support a need for continued research on the connection between vitamin D and health parameters including lipid markers and liver enzymes.

We observed the well-known relationships between LDL and other lipid markers (e.g. total cholesterol, HDL-C, and triglycerides). However, we also found a correlation between LDL and multiple biomarkers of iron stores. Excess iron directly modulates activities of several key enzymes for cholesterol and triglyceride homeostasis (e.g., 3-hydroxy-3-methylglutaryl coenzyme A reductase, cholesterol 7alpha-hydroxylase, acyl-CoA:cholesterol acyltransferase, and lipoprotein lipase), which might explain perturbations of lipid metabolism in conditions of iron overload^16^. Additionally, links between excess iron and lipid metabolism have been noted^17^, and can have downstream implications for cardiovascular risk through the oxidation of LDL^18^.

The clustering analysis reinforced expected biomarker communities, such as those related to inflammation and to iron metabolism. However, we note multiple novel findings identified by the network. First, it is established that postprandial levels of neutrophils increase after high triglyceride formulas are administered enterally and *ad libitum*^19,20^; however, there is limited evidence of the connection between plasma triglycerides and neutrophil levels outside of the postprandial window, e.g. in relation to hyperlipidemia and atherosclerosis^21^. Our findings may offer support to this relationship, since triglycerides and neutrophils are positively correlated in our population. Additionally, an unexpected finding was the grouping of magnesium levels with biomarkers of muscle stress (creatine kinase, ALT, and AST)^22^. Exercise induces a redistribution of magnesium to tissues where energy production is taking place^23^, and in the post-exercise period, magnesium is mobilized and redistributed back into circulation. The amount of muscle damage is a key factor in this release of magnesium^24^. Although magnesium has been linked to exercise performance and recovery, its relationship to muscle damage is not well-established.

The above examples demonstrate the utility of our approach as a hypothesis generation mechanism for future research, as it can be used to extract possible mechanisms for relationships between these biomarkers and health or disease outcomes in generally healthy individuals. As the amount of participating individuals in the InsideTracker program grows, we anticipate the ability to increase the complexity of this approach by examining nonlinearities in biomarker relationships and focusing specifically on biomarker changes in clinically important ranges.

To understand whether improvements in biomarkers were seen during use of the platform, we assessed longitudinal changes for individuals whose baseline values were out of the clinically acceptable range. This is the group of people (for each biomarker) who might best benefit from an improvement and who would have all received recommendations tailored toward improvement of the relevant biomarker. We observed notable improvements in most of the biomarkers analyzed. Nonetheless, through an observational analysis such as this, one cannot establish the causality of platform use on biomarker changes, or resolve which component of the intervention may have been related to the results. Weight changes are important to consider as a confounding factor, although the small magnitude of the average observed BMI change suggests that weight is only one component driving the longitudinal results.

Beyond the direct effects of the recommendations provided by the platform, two other factors may contribute to the results observed. First, improvements could simply result from the participants being informed of problematic biomarkers, inspiring lifestyle changes independent of any recommendations received. The fact that choosing any set of interventions at all correlated with greater biomarker improvements in vitamin D and LDL in out-of-range participants (Fig. 4) suggests that this phenomenon likely does not fully explain the observed changes, though this pattern did not hold in other biomarkers such as HDL (data not shown). Second, the observed effect may reflect a statistical regression to the mean, which would occur due to our non-random selection of participants based on baseline values^25^. Because these baseline values are subject to random variation (e.g., due to technical variability in test results), the chosen subset of participants would be expected to show improved follow-up test values simply by chance. Though this may also explain some of the observed effect reported in Table 2, the numerous biomarker changes observed in the full population (Supplementary Table S4), many of whom had baseline values that were normal or at the opposite end of the spectrum, do not support this effect being the primary explanatory factor.

To take advantage of our access to chosen interventions, we could assess whether particular interventions were strongly associated with changes in specific biomarkers. With a focus on vitamin D and LDL, we compared proportions of participants choosing a series of interventions in those who did and did not show biomarker changes in the desired direction. As expected, vitamin D improvement did associate nominally with choice of vitamin D supplementation as an intervention. We also observed nominal associations between LDL improvement and choice to consume more oats, green tea, and dairy, all of which were no longer statistically significant after multiple hypothesis test correction. There is a precedent in the literature for consumption of both oatmeal and green tea and reduction of LDL levels, though the evidence is mixed with respect to dairy^26–28^. We note that this analysis is meant for hypothesis generation only, as we lack metrics of intervention adherence in this dataset.

We acknowledge multiple limitations associated with this observational study. Though the sample size was the largest to our knowledge containing the same combination of data types, it was still relatively small to detect more subtle correlations between biomarkers, such as those of lower magnitude, those that only appear in some subset of the overall population, or nonlinear relationships. Furthermore, the population was self-selected and required a financial investment, which may be associated with an unusually high level of adherence to recommendations. Additionally, our dietary habits, exercise, and lifestyle questionnaire has not been validated externally, and no validated measure of compliance was available for use in this population. Future research in this area should include a larger group of participants, weight measurements concurrent with biomarker measurements, and a more heterogeneous population.

In conclusion, we have used a rich longitudinal dataset of clinical biomarkers to uncover novel biological relationships while validating known ones. Furthermore, we have demonstrated that the personalized health platform described here associates with improvements in health parameters and shows promise for the validation of biomarker-intervention associations in a “real world” setting. By tracking a variety of biomarkers in free-living individuals, this platform provides a novel resource for exploration and hypothesis generation. With the advent of personalized health platforms such as the one described here, new efforts should be devoted to understanding the efficacy of their individual components while incorporating the valuable insights of participants.

## Methods

### Dataset

We conducted an observational analysis of data from Segterra, a company established in 2009 that markets and sells InsideTracker (insidetracker.com), a personalized lifestyle recommendation platform. The platform provides serum biomarker testing, analysis, and recommendations for improving out-of-range serum biomarkers. New users were continuously added to the InsideTracker database from January 2011 to September 2017.

### Description of algorithm-based recommendation platform

The web-based platform was accessible through a computer, tablet, or mobile device. Its core was a database of over 1500 lifestyle-focused recommendations curated from peer-reviewed, scientific publications. Each participant’s recommendations were individually generated by a rule-based algorithmic expert system that used serum biomarker levels, demographic information, dietary restrictions, physical activity, dietary supplement regimen, and lifestyle parameters as inputs.

When participants logged into the InsideTracker platform, they had access to their blood work results as well as their suggested recommendations. Participants received the recommendations as short text descriptions that included the action with instruction on frequency, amount, and execution, where applicable. Exercise recommendations were provided with frequency and intensity instructions, food recommendations were provided with frequency, serving size, and recipes, supplement recommendations were provided with dosage and time of day instructions, and lifestyle recommendations were provided with detailed implementation descriptions specific to the recommended action. Each recommendation included a link to the PubMed webpage of the primary publication that supported the recommendation, as well as a grade indicator (one to five stars) that communicated the scientific quality of each recommendation. The grading system was calculated using a formula designed to minimize bias through use of the following criteria: number of supporting studies and their population sizes, impact factor of the journal, year of publication, study design, magnitude of effect of the intervention on a specific biomarker, and study outcome.

Participants created an action plan by selecting 5 out of a maximum of 20 suggested recommendations. An engagement tool encouraged compliance by sending reminders on a regular basis, and participants were encouraged to retest their biomarker panel every 3–6 months.

### Recruitment of participants

Recruitment of participants aged 18 or older and residing in North America was conducted through company marketing and outreach. Participants were subscribing members to the InsideTracker platform, and provided informed consent to have their blood test data and self-reported information used in an anonymized fashion for research purposes. Research was conducted according to guidelines for observational research in tissue samples from human subjects. Eligible participants completed a questionnaire that included age, ethnicity, sex, dietary preferences, physical activity, and exposure to sunlight. This study employed data from 1032 participants that met our analysis inclusion requirements, namely having at least two measures of at least one of the 40 biomarkers, with the follow-up blood test at least 30 days after the baseline test. The platform is not a medical service and does not diagnose or treat medical conditions, so medical history and medication use were not collected.

### Biomarker collection and analysis

Blood samples were collected and analyzed by Clinical Laboratory Improvement Amendments (CLIA)–approved, third-party clinical labs (primarily Quest Diagnostics and LabCorp). Participants were instructed to fast for 12 hours prior to the phlebotomy, with the exception of water consumption. Results from the blood analysis were then uploaded to the platform via electronic integration with the CLIA-approved lab. Participants chose a specific blood panel from 7 possible offerings, each comprising some subset of the biomarkers available. Due to the variation in blood panels offered, the participant sample size per biomarker is not uniform.

### Biomarker dataset preparation

In our raw dataset, occasional outlier values were observed that were deemed implausible (e.g. fasting glucose <20 mg/dL). To remove anomalous outliers in a systematic way, we developed a basic method for determining “plausibility cutoffs” based on the existing repository of blood biomarker data from the National Health and Nutrition Examination Survey (NHANES I, II, III, and continuous NHANES, spanning 1971–2014). For each biomarker, 0.5 and 99.5 percentiles were used as the outer limits for acceptable values. Participants whose baseline or follow-up values were outside this range were removed from the analysis of that particular biomarker.

### Correlation network construction

In order to determine which biomarkers tend to change together, a pairwise Spearman correlation test was performed for each pair of biomarkers in the dataset. The number of available observations differed for each test, because not all biomarkers examined were measured for each participant at each time point. Significance tests were performed for each correlation and participant to a correction for the 780 comparisons using the Benjamini-Hochberg procedure^29^. Network visualizations were constructed using the Cytoscape program (version 3.2.1)^30^, including only those edges representing correlations with p < 0.05 after multiple test correction.

To find correlation network submodules that may represent biological phenomena of interest, a hierarchical clustering analysis was performed. The distance metric was calculated as *d*_*ij*_ = 1 − |*cor*_*ij*_|. The dynamic tree cutting algorithm of Langfelder and Horvath was used as an unbiased method of selecting communities from the resulting dendrogram^11^. This and all subsequent analysis was performed using the R statistical program (version 3.3.2)^31^.

### Longitudinal analysis

Longitudinal changes in biomarkers between baseline and follow-up were assessed using the Wilcoxon signed-rank test for paired samples. For a subsequent analysis, the data were filtered to include only those participants whose baseline value was outside the clinically acceptable range for each biomarker. Though values can be too high or too low, here a single “direction of risk” was chosen as the most common direction in which individuals tend to be out-of-range, and the corresponding cutoffs used were based on those employed by Quest Diagnostics (Table 2). In the filtered analysis, only biomarkers with at least 20 participants out-of-range at baseline were included, and p-values shown have not been corrected for multiple hypothesis tests.

To account for the seasonal fluctuation in vitamin D levels, we performed a simple adjustment by regressing all vitamin D levels in our population (baseline and follow-up tests) on a binary “summer” variable (denoting lab tests taken from June through September). This showed an average increase of 2.5 mg/dL in vitamin D during the summer, which we used as an adjustment factor before significance testing of vitamin D in both the full and out-of-range analyses.

### Intervention choice and biomarker change analysis

To understand whether specific interventions were associated with changes in specific biomarkers, proportions of participants with an improving biomarker over time were calculated after stratifying by whether a given intervention was chosen. Statistical significance of these proportions was tested using a Chi-square test, with p-values calculated by Monte Carlo simulation. This process was performed for vitamin D and LDL across the 10 most frequently chosen interventions, with “improvement” defined as an increase of any magnitude in vitamin D or a decrease in LDL. Those participants who did not choose any intervention were included in the group not choosing each intervention. To increase the available sample size, the entire population (not just those out-of-range) was used in this analysis.

## Electronic supplementary material

Supplementary Information

Full Correlation Table

## Data Availability

The full set of biomarker change correlations has been made available in the Supplementary Information files. Specific components of the raw dataset are available upon reasonable request from the corresponding author.
