# Publisher Correction: Longitudinal analysis of biomarker data from a personalized nutrition platform in healthy subjects

**DOI:** 10.1038/s41598-018-35249-y

**Published:** 2018-11-08

**Authors:** Kenneth Westerman, Ashley Reaver, Catherine Roy, Margaret Ploch, Erin Sharoni, Bartek Nogal, David A. Sinclair, David L. Katz, Jeffrey B. Blumberg, Gil Blander

**Affiliations:** 1 InsideTracker, Cambridge, Massachusetts United States of America; 2 The Friedman School of Nutrition Science and Policy at Tufts University, Boston, Massachusetts United States of America; 3 Yale University Prevention Research Center, Griffin Hospital, Yale University School of Medicine, Derby, Connecticut United States of America; 4 Department of Genetics, Harvard Medical School, Boston, Massachusetts United States of America; 5 Department of Pharmacology, The University of New South Wales, Sydney, New South Wales Australia

Correction to: *Scientific Reports* 10.1038/s41598-018-33008-7, published online 02 October 2018

Information included in the Acknowledgements section of this Article should have been included in the Competing Interests section, which should read:-

“This study was funded by InsideTracker. The scientists who conducted this study and prepared this manuscript are either employed by the sponsoring company (K.W., A.R., C.R., M.P., E.S., B.N., G.B.) or serve as advisors to it (D.S., D.K., J.B.). Thus, there is no separation between investigative team and funder, which provided support in the form of salaries, and was involved in the study design, data collection and analysis, decision to publish, and preparation of the manuscript.”

