# Supplementary material for: Longitudinal analysis of biomarker data from a personalized nutrition platform in healthy subjects
Source: Sci Rep. 2018 Oct 2;8:14685. doi: 10.1038/s41598-018-33008-7 (PMC6168584; doi:10.1038/s41598-018-33008-7)
Supplement: Supplementary file 1 — Supplementary Information [file 41598_2018_33008_MOESM1_ESM.pdf]

Supplementary Data: Longitudinal analysis of biomarker data from a personalized nutrition platform in healthy subjects

Kenneth Westerman, Ashley Reaver, Catherine Roy, Margaret Ploch, Erin Sharoni, Bartek Nogal, David A. Sinclair, David L. Katz, Jeffrey B. Blumberg, Gil Blander

Intervention	n	% choosing
Vitamin D3	303	29.4
Psyllium Husk	240	23.3
Berberine	217	21.0
Garlic	195	18.9
Increase fiber	174	16.9
Eat more fish	164	15.9
Eat more oatmeal	162	15.7
Drink green tea	136	13.2
Intermittent fasting	134	13.0
Eat more dairy	121	11.7
Eat nuts every day	120	11.6
Spirulina	105	10.2
Go vegetarian	101	9.8
Lose weight	101	9.8
Eat more fiber	91	8.8
Vitamin C	91	8.8
Reduce red meat consumption	88	8.5
Eat enough calories	86	8.3
Get plenty of sleep	82	7.9
Eat enough protein	81	7.8

Supplementary Table S1: Most popular interventions chosen by participants.

Biomarker	Abbreviation	Measurement units
Albumin	Alb	g/L
Alanine aminotransferase	ALT	U/L
Aspartate aminotransferase	AST	U/L
Calcium	Ca	mg/dL
Cholesterol	Chol	mg/dL
Cortisol	Cor	ug/dL
Creatine kinase	CK	U/L
Ferritin	Fer	ng/mL
Folate	Fol	ng/mL
Free testosterone	FT	ng/dL
Gamma-glutamyl transferase	GGT	U/L
Glucose	Glu	mg/dL
High-density lipoprotein cholesterol	HDL	mg/dL
Hemoglobin	Hb	g/dL
High sensitivity C-reactive protein	hsCRP	mg/dL
Serum iron	FE	ug/dL
Low-density lipoprotein cholesterol	LDL	mg/dL
Magnesium	Mg	mg/dL
Potassium	K	mmol/L
Sex hormone binding globulin	SHBG	nmol/L
Sodium	Na	mmol/L
Testosterone	Tes	ng/dL
Total iron binding capacity	TIBC	ug/dL
Triglycerides	Tg	mg/dL
Vitamin B12	B12	pg/mL
Vitamin D	D	ng/mL
White blood cell count	WBC	x10e3
Red blood cells	RBC	x10e6
Neutrophils	NEUT	cells/uL
Basophils	BASOS	cells/uL
Eosinophils	EOS	cells/uL
Lymphocytes	LYMPHS	cells/uL
Monocytes	MONOS	cells/uL
Hematocrit	HCT	%
Mean corpuscular volume	MCV	fL
Mean corpuscular hemoglobin	MCH	pg
Mean corpuscular hemoglobin concentration	MCHC	g/dL
Red cell distribution width	RDW	%
Platelets	PLT	thousands/uL
Mean platelet volume	MPV	fL

Supplementary Table S2: Biomarker list.

Biomarker combination	Full population	<2 yr. re-test	Male	Female
Mg-ALT	0.096	0.126	0.080	0.125
Mg-CK	0.093	0.114	0.134	0.011
Ferritin-LDL	0.161	0.182	0.160	0.155
Triglycerides-Neutrophils	0.389	0.385	0.414	0.364

Supplementary Table S3: Sensitivity analysis for correlations. Shown are multi-biomarker Spearman correlations as determined using the full population vs. various sub-groups. Broad correlations between results (Pearson correlations between correlation coefficients in the full population and those of a sub-group) were high across strata: 0.987 for those re-testing within 2 years (n=897), 0.961 for males (n=672), and 0.878 for females (n=360).

Biomarker	Baseline median (IQR)	Follow-up median (IQR)	P-value	Sample size
Vitamin D	32.9 (17.3)	38 (15.9)	<0.001	945
Ferritin	100 (126)	91 (114.25)	<0.001	944
Vitamin B12	568.5 (323.25)	545.5 (314.75)	<0.001	846
Testosterone	397.5 (533)	446.5 (585)	<0.001	782
Low-density lipoprotein cholesterol	115 (47)	110 (45)	<0.001	928
Albumin	4.5 (0.33)	4.5 (0.4)	<0.001	464
Mean corpuscular hemoglobin	30.6 (1.6)	30.8 (1.8)	<0.001	168
Total iron binding capacity	314 (59.5)	321 (57.5)	0.002	455
Aspartate aminotransferase	23 (10)	22 (10)	0.003	474
White blood cell count	5.3 (1.7)	5.1 (1.8)	0.003	833
Sodium	140 (3)	139 (3)	0.003	803
Magnesium	2.1 (0.2)	2.1 (0.2)	0.004	889
Mean platelet volume	9.6 (1.9)	10.1 (1.7)	0.006	166
Red cell distribution width	13.4 (1)	13.1 (1.05)	0.011	168
Cortisol	14.4 (7.95)	13.7 (7)	0.027	503
Basophils	30 (18.5)	32 (22.5)	0.031	167
Mean corpuscular volume	93.1 (5.93)	93.15 (5.62)	0.078	168
Alanine aminotransferase	21 (13)	21 (12.75)	0.086	578
Serum iron	108 (47.5)	114 (51.83)	0.103	459
Gamma-glutamyl transferase	15 (9)	15 (9)	0.131	474
Free testosterone	9.7 (4.6)	9.9 (4.3)	0.139	307
Triglycerides	72 (43.25)	72 (43)	0.169	945
Folate	15.6 (7.2)	15.3 (8.07)	0.239	886
Neutrophils	2605 (1073.5)	2576 (1035)	0.262	167
Sex hormone binding globulin	49.3 (38)	53 (34.7)	0.278	477
High sensitivity C-reactive protein	0.5 (0.92)	0.5 (0.9)	0.409	590
Lymphocytes	1729 (654)	1643 (680)	0.432	167
High-density lipoprotein cholesterol	60 (23)	61 (22)	0.443	938
Hematocrit	43.7 (5.5)	43.55 (5.12)	0.46	168
Mean corpuscular hemoglobin concentration	33 (1.32)	33 (1.3)	0.46	168
Monocytes	384 (211)	393 (150.5)	0.556	167
Creatine kinase	136.5 (142)	133.5 (124)	0.624	914
Calcium	9.5 (0.4)	9.5 (0.4)	0.667	904
Eosinophils	100 (134.5)	111 (99.5)	0.71	167
Hemoglobin	14.5 (1.6)	14.5 (1.6)	0.764	938
Cholesterol	188 (48.5)	187 (51)	0.779	955
Potassium	4.4 (0.4)	4.3 (0.5)	0.787	802
Red blood cells	4.67 (0.69)	4.68 (0.62)	0.89	169
Platelets	217 (59.5)	219 (62)	0.956	167
Glucose	88 (11)	88 (11)	0.993	955

Supplementary Table S4: Longitudinal results for the full set of available changes over time (no filtering for out-of-range participants).

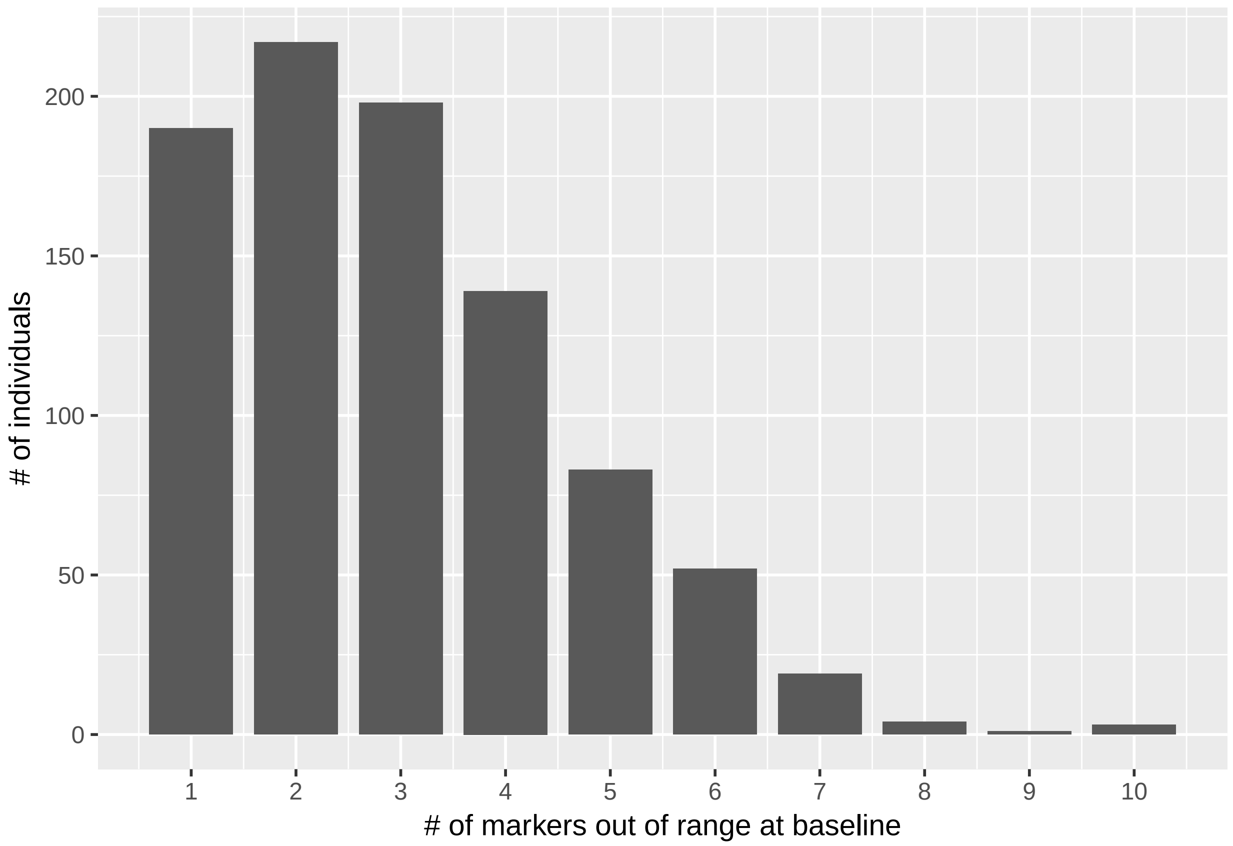

Supplementary Figure S1: Distribution of number of out-of-range markers per participant at baseline.
