# Supplementary material for: Longitudinal analysis of biomarker data from a personalized nutrition platform in healthy subjects
Source: Sci Rep. 2018 Oct 2;8:14685. doi: 10.1038/s41598-018-33008-7 (PMC6168584; doi:10.1038/s41598-018-33008-7)
Supplement: Supplementary file 2 — Full Correlation Table [file 41598_2018_33008_MOESM2_ESM.pdf]

BM1	BM2	corr	p	n
Alb	Alb	1	0	464
ALT	Alb	0.02107112	0.67476208	399
AST	Alb	0.15742491	6.94E-04	461
B12	Alb	0.18517267	7.08E-05	455
BASOS	Alb	-0.0858524	0.33719867	127
Ca	Alb	0.44347574	0	461
Chol	Alb	0.36724554	4.44E-16	460
CK	Alb	0.11520926	0.0146939	448
Cor	Alb	-0.0267449	0.57019616	453
D	Alb	-0.0360362	0.44776382	446
EOS	Alb	0.04905347	0.58391947	127
FE	Alb	0.11756297	0.01199522	456
Fer	Alb	0.26821539	4.89E-09	461
Fol	Alb	0.11179885	0.01717047	454
FT	Alb	-0.0345084	0.55903606	289
GGT	Alb	0.12552272	0.00696631	461
Glu	Alb	-0.0010235	0.98261033	456
Hb	Alb	0.3709026	4.44E-16	448
HCT	Alb	0.38262426	9.02E-06	127
HDL	Alb	0.26283558	1.13E-08	458
hsCRP	Alb	-0.0635923	0.17665905	453
K	Alb	0.04872429	0.30130532	452
LDL	Alb	0.33829342	1.65E-13	450
LYMPHS	Alb	0.10767004	0.22824624	127
MCH	Alb	-0.0842207	0.34649523	127
MCHC	Alb	-0.0646167	0.47044908	127
MCV	Alb	0.04215038	0.63798123	127
Mg	Alb	0.08808234	0.05990573	457
MONOS	Alb	0.03786668	0.67253742	127
MPV	Alb	0.05194453	0.56508056	125
Na	Alb	0.10412383	0.02685677	452
NEUT	Alb	0.21245002	0.01648671	127
PLT	Alb	0.16792597	0.06016967	126
RBC	Alb	0.44905935	1.06E-07	128
RDW	Alb	-0.0090025	0.91998575	127
SHBG	Alb	0.06973117	0.14466406	439
Tes	Alb	-0.0721317	0.12739372	448
Tg	Alb	0.07866164	0.09196429	460
TIBC	Alb	0.20943302	7.12E-06	452
WBC	Alb	0.10068484	0.03292891	449
Alb	ALT	0.02107112	0.8434526	399
ALT	ALT	1	0	578

AST	ALT	0.68491827	0	410
B12	ALT	0.14229232	8.84E-04	543
BASOS	ALT	0.00797261	0.92578566	139
Ca	ALT	0.06675826	0.13179093	511
Chol	ALT	0.03867737	0.37692207	524
CK	ALT	0.28106674	3.31E-11	537
Cor	ALT	0.02742083	0.57248006	426
D	ALT	-0.03269	0.4442064	550
EOS	ALT	0.15007709	0.077832	139
FE	ALT	0.00156316	0.97529442	395
Fer	ALT	0.11576402	0.00632853	555
Fol	ALT	-0.0038898	0.93058389	504
FT	ALT	-0.0688947	0.27688599	251
GGT	ALT	0.30347037	3.68E-10	409
Glu	ALT	0.10348876	0.01664143	535
Hb	ALT	0.04642881	0.27881588	546
HCT	ALT	-0.1792471	0.03408542	140
HDL	ALT	0.04092234	0.35122455	521
hsCRP	ALT	0.14436575	0.00110266	508
K	ALT	0.14031939	0.00144161	513
LDL	ALT	-0.0284751	0.51948918	514
LYMPHS	ALT	0.05256727	0.53882717	139
MCH	ALT	0.07187977	0.39869161	140
MCHC	ALT	0.17838949	0.03496579	140
MCV	ALT	-0.1283161	0.13081446	140
Mg	ALT	0.09574817	0.03111787	507
MONOS	ALT	0.14899808	0.08001762	139
MPV	ALT	0.02881698	0.73723782	138
Na	ALT	0.00386417	0.93035844	514
NEUT	ALT	0.11353245	0.18327207	139
PLT	ALT	0.14634321	0.0856056	139
RBC	ALT	-0.0445881	0.60090193	140
RDW	ALT	-0.2049043	0.01515933	140
SHBG	ALT	0.03732355	0.45047471	411
Tes	ALT	-0.0613425	0.16785854	507
Tg	ALT	0.11754428	0.00712285	523
TIBC	ALT	0.0172675	0.73357419	391
WBC	ALT	0.08468949	0.05068583	533
Alb	AST	0.15742491	0.00555336	461
ALT	AST	0.68491827	0	410
AST	AST	1	0	474
B12	AST	0.10772026	0.02155486	455
BASOS	AST	0.03451022	0.70125282	126

Ca	AST	0.03262726	0.4851405	460
Chol	AST	-0.01847	0.68929235	471
CK	AST	0.62418044	0	447
Cor	AST	-0.0035939	0.93926345	452
D	AST	-0.0363645	0.44006032	453
EOS	AST	0.14050828	0.11658307	126
FE	AST	0.0203638	0.66485148	455
Fer	AST	0.1301261	0.00480945	468
Fol	AST	0.05485505	0.243948	453
FT	AST	-0.0793223	0.17719531	291
GGT	AST	0.24836717	4.72E-08	471
Glu	AST	-0.0178669	0.70046921	466
Hb	AST	-0.0010143	0.98274716	457
HCT	AST	-0.1107322	0.21706192	126
HDL	AST	0.11055084	0.01661625	469
hsCRP	AST	0.1590171	6.82E-04	453
K	AST	0.15974945	5.68E-04	462
LDL	AST	-0.0508772	0.27565988	461
LYMPHS	AST	-0.1022329	0.2546564	126
MCH	AST	0.00887292	0.92144981	126
MCHC	AST	0.02127918	0.81304126	126
MCV	AST	-0.0019118	0.98304921	126
Mg	AST	0.15112282	0.00120856	456
MONOS	AST	0.01047472	0.90732774	126
MPV	AST	0.16190397	0.07241513	124
Na	AST	0.01354278	0.77133323	463
NEUT	AST	-0.0860206	0.33819782	126
PLT	AST	-0.0029188	0.97422823	125
RBC	AST	-0.0987022	0.26958528	127
RDW	AST	-0.1261119	0.15939204	126
SHBG	AST	0.05068419	0.28932433	439
Tes	AST	-0.0753876	0.10828823	455
Tg	AST	-0.1121134	0.01491824	471
TIBC	AST	0.04371033	0.35437721	451
WBC	AST	0.00335925	0.94271957	460
Alb	B12	0.18517267	7.68E-04	455
ALT	B12	0.14229232	0.00696532	543
AST	B12	0.10772026	0.09137386	455
B12	B12	1	0	847
BASOS	B12	-0.1802375	0.02529831	154
Ca	B12	0.12859246	2.63E-04	801
Chol	B12	0.04584275	0.19494309	801
CK	B12	0.04625433	0.18442578	825

Cor	B12	-0.0075234	0.86806647	490
D	B12	0.1305798	1.92E-04	811
EOS	B12	-0.1102634	0.17340634	154
FE	B12	0.01565659	0.73852785	457
Fer	B12	0.17900925	1.65E-07	844
Fol	B12	0.11397045	0.00125081	799
FT	B12	0.0139305	0.80854888	305
GGT	B12	0.01942508	0.67942328	455
Glu	B12	-0.0544745	0.12462678	796
Hb	B12	0.11151035	0.0013095	828
HCT	B12	0.04104154	0.61212623	155
HDL	B12	0.09821728	0.00588514	785
hsCRP	B12	-0.0268902	0.52652447	557
K	B12	0.06374085	0.07578173	777
LDL	B12	0.0521494	0.14720286	774
LYMPHS	B12	0.14168121	0.07964568	154
MCH	B12	0.1359122	0.0917515	155
MCHC	B12	0.0276511	0.73270299	155
MCV	B12	0.02320959	0.77437525	155
Mg	B12	0.05235443	0.13900508	800
MONOS	B12	-0.0980671	0.22628487	154
MPV	B12	-0.0944472	0.2455342	153
Na	B12	0.05672509	0.11365841	779
NEUT	B12	-0.1238001	0.12609297	154
PLT	B12	0.09614162	0.23557674	154
RBC	B12	0.02786683	0.73069754	155
RDW	B12	-0.0320749	0.69195837	155
SHBG	B12	0.03713473	0.42037666	473
Tes	B12	-0.1169822	0.00134022	749
Tg	B12	-0.0535563	0.13258157	790
TIBC	B12	-0.1240229	0.00822735	453
WBC	B12	0.01749697	0.6233946	790
Alb	BASOS	-0.0858524	0.59104485	127
ALT	BASOS	0.00797261	0.96651855	139
AST	BASOS	0.03451022	0.85465188	126
B12	BASOS	-0.1802375	0.10552235	154
BASOS	BASOS	1	0	167
Ca	BASOS	0.13422393	0.10385869	148
Chol	BASOS	-0.0080647	0.92276937	147
CK	BASOS	-0.0295761	0.706958	164
Cor	BASOS	-0.0164546	0.84644064	141
D	BASOS	-0.0701173	0.37980735	159
EOS	BASOS	0.07935341	0.30802341	167

FE	BASOS	-0.1187334	0.18192066	128
Fer	BASOS	-0.0630055	0.41999118	166
Fol	BASOS	-0.0346311	0.67605053	148
FT	BASOS	0.0644183	0.57779995	77
GGT	BASOS	-0.0277579	0.75672683	127
Glu	BASOS	0.05808191	0.48317949	148
Hb	BASOS	-0.0632436	0.41966013	165
HCT	BASOS	-0.0900696	0.24704027	167
HDL	BASOS	-0.0412683	0.62090292	146
hsCRP	BASOS	-0.0073897	0.92800514	152
K	BASOS	0.04205769	0.62556535	137
LDL	BASOS	0.04826884	0.56152956	147
LYMPHS	BASOS	-0.0015647	0.98398905	167
MCH	BASOS	0.05521167	0.47852692	167
MCHC	BASOS	0.08303743	0.28603329	167
MCV	BASOS	-0.0342942	0.65995227	167
Mg	BASOS	-0.1184035	0.15037988	149
MONOS	BASOS	0.21836086	0.00458339	167
MPV	BASOS	0.25405099	9.92E-04	165
Na	BASOS	0.05746944	0.50473969	137
NEUT	BASOS	0.10232814	0.18820904	167
PLT	BASOS	-0.0467053	0.55015073	166
RBC	BASOS	-0.0761636	0.32793602	167
RDW	BASOS	-0.3174217	2.92E-05	167
SHBG	BASOS	-0.1741979	0.04101292	138
Tes	BASOS	-0.0078413	0.9236182	152
Tg	BASOS	-0.0483722	0.56069082	147
TIBC	BASOS	-0.0475421	0.59410131	128
WBC	BASOS	0.07517343	0.34478192	160
Alb	Ca	0.44347574	0	461
ALT	Ca	0.06675826	0.33685219	511
AST	Ca	0.03262726	0.72766479	460
B12	Ca	0.12859246	0.00250526	801
BASOS	Ca	0.13422393	0.29009771	148
Ca	Ca	1	0	905
Chol	Ca	0.30548724	0	896
CK	Ca	-0.1208894	3.49E-04	871
Cor	Ca	0.02678513	0.56705757	459
D	Ca	0.04018611	0.23827595	863
EOS	Ca	0.05966069	0.47134551	148
FE	Ca	0.14512584	0.0018682	457
Fer	Ca	0.13844562	3.44E-05	889
Fol	Ca	0.11965734	3.69E-04	882

FT	Ca	0.08229577	0.16145509	291
GGT	Ca	0.19340362	2.96E-05	460
Glu	Ca	0.06160252	0.06576181	893
Hb	Ca	0.33116799	0	882
HCT	Ca	0.21978851	0.00727311	148
HDL	Ca	0.18644854	2.52E-08	880
hsCRP	Ca	-0.0149342	0.73330395	523
K	Ca	0.22168877	2.98E-10	790
LDL	Ca	0.2136836	2.02E-10	868
LYMPHS	Ca	0.04738032	0.56743008	148
MCH	Ca	0.08349052	0.31304343	148
MCHC	Ca	0.11091148	0.17959596	148
MCV	Ca	-0.0313786	0.70498949	148
Mg	Ca	0.13722925	4.16E-05	886
MONOS	Ca	0.04267961	0.60651653	148
MPV	Ca	0.08765905	0.29274874	146
Na	Ca	0.13872451	9.25E-05	789
NEUT	Ca	0.17086528	0.03786295	148
PLT	Ca	0.07604753	0.35993828	147
RBC	Ca	0.31112178	1.12E-04	149
RDW	Ca	-0.1167766	0.15752519	148
SHBG	Ca	0.05764638	0.22646324	442
Tes	Ca	0.07673987	0.04036639	714
Tg	Ca	0.10255099	0.00224118	886
TIBC	Ca	0.24500136	1.29E-07	453
WBC	Ca	0.10340735	0.00357565	792
Alb	Chol	0.36724554	2.31E-14	460
ALT	Chol	0.03867737	0.63225638	524
AST	Chol	-0.01847	0.85172962	471
B12	Chol	0.04584275	0.41659071	801
BASOS	Chol	-0.0080647	0.96644795	147
Ca	Chol	0.30548724	0	896
Chol	Chol	1	0	956
CK	Chol	-0.0701687	0.03863429	869
Cor	Chol	0.038011	0.41198746	468
D	Chol	-0.0550537	0.10207846	883
EOS	Chol	0.09632214	0.2458148	147
FE	Chol	0.06586532	0.16073195	455
Fer	Chol	0.14002533	2.57E-05	897
Fol	Chol	0.04123994	0.22164884	880
FT	Chol	-0.0609946	0.29641964	295
GGT	Chol	0.20352233	8.70E-06	470
Glu	Chol	0.05786536	0.07873954	924

Hb	Chol	0.26344957	1.33E-15	891
HCT	Chol	0.19770016	0.01638322	147
HDL	Chol	0.40199454	0	937
hsCRP	Chol	-0.026525	0.54116905	533
K	Chol	0.08261282	0.01990267	794
LDL	Chol	0.83601357	0	927
LYMPHS	Chol	0.12292917	0.13798269	147
MCH	Chol	-0.016246	0.84515546	147
MCHC	Chol	0.02998694	0.71843612	147
MCV	Chol	-0.0564085	0.49737909	147
Mg	Chol	0.03297565	0.32797339	882
MONOS	Chol	-0.0046307	0.95560883	147
MPV	Chol	-0.1217099	0.14475138	145
Na	Chol	-0.0552583	0.1192878	796
NEUT	Chol	0.05608634	0.49984288	147
PLT	Chol	0.10437601	0.20992664	146
RBC	Chol	0.2313729	0.00480739	147
RDW	Chol	0.00914369	0.91247503	147
SHBG	Chol	0.00467744	0.92179649	443
Tes	Chol	0.00328544	0.92967822	724
Tg	Chol	0.30602501	0	945
TIBC	Chol	0.27320845	3.67E-09	451
WBC	Chol	0.10090075	0.0042322	802
Alb	CK	0.11520926	0.06946206	448
ALT	CK	0.28106674	1.23E-09	537
AST	CK	0.62418044	0	447
B12	CK	0.04625433	0.40636189	825
BASOS	CK	-0.0295761	0.85512048	164
Ca	CK	-0.1208894	0.00306039	871
Chol	CK	-0.0701687	0.14557849	869
CK	CK	1	0	915
Cor	CK	0.01592735	0.72697325	483
D	CK	0.01308401	0.69847617	879
EOS	CK	0.06030673	0.44302546	164
FE	CK	0.02080143	0.65987542	450
Fer	CK	0.01526272	0.64546923	911
Fol	CK	0.0389387	0.25180046	868
FT	CK	-0.1116504	0.05459983	297
GGT	CK	0.06827971	0.14906111	448
Glu	CK	-0.0875031	0.01011787	863
Hb	CK	-0.1396075	2.72E-05	897
HCT	CK	-0.2064214	0.0078121	165
HDL	CK	0.06710711	0.05021547	852

hsCRP	CK	0.1498081	4.18E-04	551
K	CK	0.04767825	0.1883153	763
LDL	CK	-0.0426948	0.21640846	840
LYMPHS	CK	-0.0294848	0.70782173	164
MCH	CK	0.02914108	0.71022261	165
MCHC	CK	-0.0110667	0.8878079	165
MCV	CK	0.02480699	0.75179174	165
Mg	CK	0.09340974	0.00580052	871
MONOS	CK	-0.0541398	0.49111922	164
MPV	CK	0.07467022	0.34347976	163
Na	CK	0.01987391	0.58336205	764
NEUT	CK	-0.1509033	0.05375775	164
PLT	CK	-0.0783041	0.31893775	164
RBC	CK	-0.2782369	2.96E-04	165
RDW	CK	0.06055933	0.43970323	165
SHBG	CK	0.04496233	0.33280156	466
Tes	CK	-0.0954974	0.00943614	738
Tg	CK	-0.1855939	4.35E-08	858
TIBC	CK	-0.0288315	0.54365048	446
WBC	CK	-0.0438793	0.21884565	787
Alb	Cor	-0.0267449	0.78100765	453
ALT	Cor	0.02742083	0.7820218	426
AST	Cor	-0.0035939	0.96908134	452
B12	Cor	-0.0075234	0.93752823	490
BASOS	Cor	-0.0164546	0.92858467	141
Ca	Cor	0.02678513	0.78059165	459
Chol	Cor	0.038011	0.66808777	468
CK	Cor	0.01592735	0.86364788	483
Cor	Cor	1	0	503
D	Cor	0.02871662	0.52809533	485
EOS	Cor	0.05432607	0.52229175	141
FE	Cor	0.00585781	0.90094238	454
Fer	Cor	0.03859242	0.39253226	493
Fol	Cor	-0.0261022	0.57740406	458
FT	Cor	0.16862091	0.00323849	303
GGT	Cor	-0.0080713	0.86367635	455
Glu	Cor	0.03389746	0.47073976	455
Hb	Cor	0.04754359	0.2970593	483
HCT	Cor	0.02293889	0.78641561	142
HDL	Cor	0.06951553	0.1352907	463
hsCRP	Cor	0.0723816	0.10632234	499
K	Cor	0.11156448	0.01753113	453
LDL	Cor	0.06990083	0.1356881	457

LYMPHS	Cor	0.13743269	0.10413764	141
MCH	Cor	-0.0589288	0.48604276	142
MCHC	Cor	0.02201965	0.79478149	142
MCV	Cor	-0.0558828	0.50890059	142
Mg	Cor	0.0537739	0.24920852	461
MONOS	Cor	0.08882098	0.29492714	141
MPV	Cor	0.03287637	0.69978172	140
Na	Cor	0.00854723	0.85603933	453
NEUT	Cor	-0.0546268	0.51998821	141
PLT	Cor	0.22014309	0.00871476	141
RBC	Cor	0.07149314	0.39615028	143
RDW	Cor	-0.0663099	0.43300898	142
SHBG	Cor	-0.008086	0.86062259	474
Tes	Cor	0.04746105	0.29890739	481
Tg	Cor	0.08988473	0.05224001	467
TIBC	Cor	0.00140679	0.97620587	452
WBC	Cor	0.03727543	0.42410933	462
Alb	D	-0.0360362	0.69851156	446
ALT	D	-0.03269	0.69574496	550
AST	D	-0.0363645	0.69483209	453
B12	D	0.1305798	0.00189883	811
BASOS	D	-0.0701173	0.63483433	159
Ca	D	0.04018611	0.47900836	863
Chol	D	-0.0550537	0.28901331	883
CK	D	0.01308401	0.85465188	879
Cor	D	0.02871662	0.75580616	485
D	D	1	0	946
EOS	D	-0.137969	0.08286496	159
FE	D	0.03412867	0.4731782	444
Fer	D	-0.0526336	0.11298323	908
Fol	D	0.09564139	0.00517982	853
FT	D	0.06882426	0.24021929	293
GGT	D	-0.1367925	0.00349606	454
Glu	D	-0.0710958	0.03445448	885
Hb	D	0.02425679	0.46909065	893
HCT	D	0.01254929	0.87485166	160
HDL	D	0.09501947	0.00515962	865
hsCRP	D	0.00973723	0.81688047	568
K	D	0.02301087	0.52646889	760
LDL	D	-0.0494735	0.14787236	857
LYMPHS	D	-0.0253185	0.75140597	159
MCH	D	0.04884412	0.53964056	160
MCHC	D	0.03961535	0.61893152	160

MCV	D	-0.0143935	0.85664511	160
Mg	D	-0.0134304	0.69460467	857
MONOS	D	-0.0391287	0.62435456	159
MPV	D	0.12077038	0.13064981	158
Na	D	-0.0060926	0.86674306	761
NEUT	D	-0.2046092	0.00967884	159
PLT	D	-0.130057	0.10226625	159
RBC	D	-0.0316337	0.6903659	161
RDW	D	-0.0475172	0.55072442	160
SHBG	D	-0.0761231	0.10335513	459
Tes	D	0.01429724	0.69547524	752
Tg	D	-0.1224613	2.89E-04	872
TIBC	D	-0.0873056	0.06730616	440
WBC	D	-0.0524961	0.14093646	788
Alb	EOS	0.04905347	0.78727224	127
ALT	EOS	0.15007709	0.23995636	139
AST	EOS	0.14050828	0.31465327	126
B12	EOS	-0.1102634	0.39433512	154
BASOS	EOS	0.07935341	0.55316443	167
Ca	EOS	0.05966069	0.72226809	148
Chol	EOS	0.09632214	0.48418067	147
CK	EOS	0.06030673	0.69529147	164
Cor	EOS	0.05432607	0.75208683	141
D	EOS	-0.137969	0.24859489	159
EOS	EOS	1	0	167
FE	EOS	0.02790987	0.75448775	128
Fer	EOS	0.10296192	0.18681543	166
Fol	EOS	-0.2362917	0.00383654	148
FT	EOS	-0.1970465	0.08585516	77
GGT	EOS	0.24627008	0.00525451	127
Glu	EOS	0.01783075	0.82969075	148
Hb	EOS	0.014503	0.85331697	165
HCT	EOS	-0.0336312	0.66612736	167
HDL	EOS	-0.0791174	0.34249305	146
hsCRP	EOS	0.17477549	0.03127206	152
K	EOS	-0.0476321	0.58045182	137
LDL	EOS	0.12758167	0.12357488	147
LYMPHS	EOS	0.18283091	0.018034	167
MCH	EOS	0.01996136	0.79791562	167
MCHC	EOS	0.01484006	0.84903808	167
MCV	EOS	-0.0353425	0.65023441	167
Mg	EOS	0.02192691	0.79067835	149
MONOS	EOS	0.14998683	0.05303237	167

MPV	EOS	0.04024733	0.60776664	165
Na	EOS	0.02130276	0.8048418	137
NEUT	EOS	0.00331844	0.96605095	167
PLT	EOS	-0.0661979	0.39678001	166
RBC	EOS	-0.0513739	0.50967333	167
RDW	EOS	-0.0252723	0.74579524	167
SHBG	EOS	0.11181195	0.19167373	138
Tes	EOS	-0.0275671	0.73602029	152
Tg	EOS	0.08725867	0.29329096	147
TIBC	EOS	0.01759907	0.84369235	128
WBC	EOS	0.21396975	0.00659075	160
Alb	FE	0.11756297	0.05921689	456
ALT	FE	0.00156316	0.98778824	395
AST	FE	0.0203638	0.83938504	455
B12	FE	0.01565659	0.86364788	457
BASOS	FE	-0.1187334	0.40496377	128
Ca	FE	0.14512584	0.01265541	457
Chol	FE	0.06586532	0.37312773	455
CK	FE	0.02080143	0.83837585	450
Cor	FE	0.00585781	0.95480307	454
D	FE	0.03412867	0.72226809	444
EOS	FE	0.02790987	0.87314606	128
FE	FE	1	0	459
Fer	FE	0.16744328	3.15E-04	459
Fol	FE	0.06702026	0.15305024	456
FT	FE	-0.0275108	0.64139786	289
GGT	FE	0.0378802	0.41968419	456
Glu	FE	-0.0135709	0.77379423	451
Hb	FE	0.10760174	0.02243858	450
HCT	FE	-0.0303401	0.73387907	128
HDL	FE	0.03101076	0.51030587	453
hsCRP	FE	-0.0759481	0.10606806	454
K	FE	0.02164067	0.6459636	453
LDL	FE	0.04509178	0.34261072	445
LYMPHS	FE	0.02208648	0.80455306	128
MCH	FE	-0.2202697	0.01247604	128
MCHC	FE	0.108563	0.22253685	128
MCV	FE	-0.181632	0.04018198	128
Mg	FE	0.03470431	0.45826245	459
MONOS	FE	-0.1707902	0.05391705	128
MPV	FE	-0.1352883	0.13093486	126
Na	FE	-0.0603926	0.19949052	453
NEUT	FE	0.02636955	0.76764081	128

PLT	FE	-0.0433151	0.62871425	127
RBC	FE	0.13620861	0.12376596	129
RDW	FE	-0.0134501	0.88022429	128
SHBG	FE	-0.0676172	0.15726889	439
Tes	FE	0.02109968	0.65675562	446
Tg	FE	0.01165145	0.80424381	455
TIBC	FE	0.04419042	0.34697118	455
WBC	FE	0.03078985	0.5170889	445
Alb	Fer	0.26821539	1.32E-07	461
ALT	Fer	0.11576402	0.03500889	555
AST	Fer	0.1301261	0.02885667	468
B12	Fer	0.17900925	3.21E-06	844
BASOS	Fer	-0.0630055	0.67467859	166
Ca	Fer	0.13844562	4.13E-04	889
Chol	Fer	0.14002533	3.28E-04	897
CK	Fer	0.01526272	0.83281258	911
Cor	Fer	0.03859242	0.64457929	493
D	Fer	-0.0526336	0.30813608	908
EOS	Fer	0.10296192	0.40931471	166
FE	Fer	0.16744328	0.00278777	459
Fer	Fer	1	0	945
Fol	Fer	0.00907597	0.78744903	885
FT	Fer	-0.0497628	0.38568508	306
GGT	Fer	0.1216464	0.00835949	469
Glu	Fer	-0.017934	0.59291735	891
Hb	Fer	0.07893855	0.01657003	921
HCT	Fer	0.00351333	0.96405841	167
HDL	Fer	0.06779782	0.04436102	880
hsCRP	Fer	0.01137315	0.78790935	562
K	Fer	0.00673073	0.85074984	784
LDL	Fer	0.16110693	1.83E-06	868
LYMPHS	Fer	0.33245104	1.21E-05	166
MCH	Fer	-0.117059	0.13192393	167
MCHC	Fer	0.0550188	0.48006722	167
MCV	Fer	-0.079399	0.30774485	167
Mg	Fer	0.02202663	0.51283971	885
MONOS	Fer	0.12036359	0.12242383	166
MPV	Fer	-0.1493286	0.05557848	165
Na	Fer	0.00339583	0.92432073	785
NEUT	Fer	0.02094435	0.78882293	166
PLT	Fer	0.03340738	0.66916749	166
RBC	Fer	0.09259527	0.23256492	168
RDW	Fer	-0.128924	0.09681622	167

SHBG	Fer	0.10203479	0.02600776	476
Tes	Fer	-0.1136827	0.00167124	762
Tg	Fer	0.02587846	0.4416955	886
TIBC	Fer	-0.197565	2.19E-05	455
WBC	Fer	0.03010866	0.39124241	813
Alb	Fol	0.11179885	0.07653123	454
ALT	Fol	-0.0038898	0.96651855	504
AST	Fol	0.05485505	0.48418067	453
B12	Fol	0.11397045	0.00911808	799
BASOS	Fol	-0.0346311	0.84371107	148
Ca	Fol	0.11965734	0.00318521	882
Chol	Fol	0.04123994	0.46102959	880
CK	Fol	0.0389387	0.48735573	868
Cor	Fol	-0.0261022	0.7851637	458
D	Fol	0.09564139	0.0303779	853
EOS	Fol	-0.2362917	0.02413307	148
FE	Fol	0.06702026	0.36619382	456
Fer	Fol	0.00907597	0.89691237	885
Fol	Fol	1	0	887
FT	Fol	-0.0179488	0.76085304	290
GGT	Fol	-0.0201761	0.6681005	454
Glu	Fol	-0.0024926	0.94140888	872
Hb	Fol	0.05409403	0.11084244	870
HCT	Fol	-0.1649156	0.04517619	148
HDL	Fol	0.05813139	0.08769623	864
hsCRP	Fol	-0.0270624	0.5376708	521
K	Fol	0.03570038	0.32154744	773
LDL	Fol	0.03047544	0.37429921	852
LYMPHS	Fol	-0.1140474	0.16752827	148
MCH	Fol	0.09429745	0.25428907	148
MCHC	Fol	0.20273935	0.01346463	148
MCV	Fol	-0.1645688	0.04563648	148
Mg	Fol	0.06260033	0.06327352	881
MONOS	Fol	-0.0097738	0.90614833	148
MPV	Fol	0.05094975	0.5413761	146
Na	Fol	-0.045654	0.20423965	775
NEUT	Fol	-0.1240124	0.13317807	148
PLT	Fol	-0.1254108	0.13014587	147
RBC	Fol	-0.033354	0.68634422	149
RDW	Fol	-0.207764	0.01128206	148
SHBG	Fol	0.04644216	0.33053218	441
Tes	Fol	0.03437352	0.36109861	708
Tg	Fol	-0.0791056	0.01961749	870

TIBC	Fol	-0.0031307	0.94707861	452
WBC	Fol	-0.0776383	0.03079338	774
Alb	FT	-0.0345084	0.77675523	289
ALT	FT	-0.0688947	0.52420164	251
AST	FT	-0.0793223	0.39945765	291
B12	FT	0.0139305	0.90613236	305
BASOS	FT	0.0644183	0.7851637	77
Ca	FT	0.08229577	0.37369426	291
Chol	FT	-0.0609946	0.54010782	295
CK	FT	-0.1116504	0.18356841	297
Cor	FT	0.16862091	0.02087621	303
D	FT	0.06882426	0.48167365	293
EOS	FT	-0.1970465	0.25559933	77
FE	FT	-0.0275108	0.83104707	289
Fer	FT	-0.0497628	0.64280847	306
Fol	FT	-0.0179488	0.87790736	290
FT	FT	1	0	307
GGT	FT	-0.0347459	0.55495764	291
Glu	FT	0.06918115	0.24269047	287
Hb	FT	0.20789849	2.75E-04	302
HCT	FT	0.06265986	0.58824769	77
HDL	FT	-0.0324342	0.57964185	294
hsCRP	FT	-0.0586968	0.30849555	303
K	FT	0.06108679	0.30154127	288
LDL	FT	-0.0632785	0.28618877	286
LYMPHS	FT	0.02355656	0.83885743	77
MCH	FT	0.0363725	0.75348235	77
MCHC	FT	0.173018	0.1323838	77
MCV	FT	-0.1503671	0.19178382	77
Mg	FT	-0.0271834	0.64364849	292
MONOS	FT	0.12860354	0.26499203	77
MPV	FT	0.04363259	0.70821516	76
Na	FT	-0.0830984	0.15956761	288
NEUT	FT	0.07604173	0.51098667	77
PLT	FT	0.15219492	0.18637687	77
RBC	FT	0.24480807	0.03188794	77
RDW	FT	-0.1635241	0.15530711	77
SHBG	FT	-0.1613828	0.00472217	305
Tes	FT	0.66313743	0	304
Tg	FT	-0.001228	0.98327267	294
TIBC	FT	0.0246102	0.67748642	288
WBC	FT	0.03662674	0.53020403	296
Alb	GGT	0.12552272	0.03799804	461

ALT	GGT	0.30347037	1.10E-08	409
AST	GGT	0.24836717	9.94E-07	471
B12	GGT	0.01942508	0.84521556	455
BASOS	GGT	-0.0277579	0.87443989	127
Ca	GGT	0.19340362	3.61E-04	460
Chol	GGT	0.20352233	1.26E-04	470
CK	GGT	0.06827971	0.35996181	448
Cor	GGT	-0.0080713	0.9369507	455
D	GGT	-0.1367925	0.02235183	454
EOS	GGT	0.24627008	0.03058594	127
FE	GGT	0.0378802	0.67467859	456
Fer	GGT	0.1216464	0.04376109	469
Fol	GGT	-0.0201761	0.84050023	454
FT	GGT	-0.0347459	0.7743595	291
GGT	GGT	1	0	474
Glu	GGT	0.07035118	0.12939956	466
Hb	GGT	0.12340934	0.00819395	458
HCT	GGT	0.17891767	0.0441531	127
HDL	GGT	0.1063116	0.02143444	468
hsCRP	GGT	0.17468795	1.84E-04	454
K	GGT	-0.011076	0.81232133	462
LDL	GGT	0.10716775	0.02151275	460
LYMPHS	GGT	0.03248161	0.71695931	127
MCH	GGT	-0.0582573	0.51531252	127
MCHC	GGT	-0.0355355	0.69164006	127
MCV	GGT	-0.0073963	0.93422592	127
Mg	GGT	0.02092855	0.65543486	457
MONOS	GGT	-0.0022198	0.9802398	127
MPV	GGT	-0.1334562	0.1378775	125
Na	GGT	0.01276297	0.78439386	462
NEUT	GGT	0.11637529	0.19259259	127
PLT	GGT	0.20400368	0.0219481	126
RBC	GGT	0.22863041	0.00943821	128
RDW	GGT	-0.0131788	0.88308854	127
SHBG	GGT	-0.0573195	0.22964245	441
Tes	GGT	-0.0761112	0.10378817	458
Tg	GGT	0.22060868	1.37E-06	470
TIBC	GGT	0.26938736	5.47E-09	454
WBC	GGT	0.11616084	0.01266551	460
Alb	Glu	-0.0010235	0.98778824	456
ALT	Glu	0.10348876	0.07503073	535
AST	Glu	-0.0178669	0.85465188	466
B12	Glu	-0.0544745	0.32952164	796

BASOS	Glu	0.05808191	0.7261657	148
Ca	Glu	0.06160252	0.21108729	893
Chol	Glu	0.05786536	0.24172619	924
CK	Glu	-0.0875031	0.05091571	863
Cor	Glu	0.03389746	0.72226809	455
D	Glu	-0.0710958	0.13304205	885
EOS	Glu	0.01783075	0.92153619	148
FE	Glu	-0.0135709	0.88694962	451
Fer	Glu	-0.017934	0.79055647	891
Fol	Glu	-0.0024926	0.97001179	872
FT	Glu	0.06918115	0.48413956	287
GGT	Glu	0.07035118	0.33685219	466
Glu	Glu	1	0	956
Hb	Glu	0.05704993	0.08985589	885
HCT	Glu	0.01691135	0.83834914	148
HDL	Glu	-0.0175806	0.59675759	908
hsCRP	Glu	-0.0458841	0.288975	536
K	Glu	0.10071487	0.00455242	792
LDL	Glu	0.04453519	0.18289857	896
LYMPHS	Glu	-0.0586264	0.47907993	148
MCH	Glu	-0.037468	0.65118821	148
MCHC	Glu	0.05301879	0.52218378	148
MCV	Glu	-0.0589096	0.47695556	148
Mg	Glu	-0.0314725	0.35216353	876
MONOS	Glu	-0.1073045	0.19425336	148
MPV	Glu	0.10191854	0.22091775	146
Na	Glu	-0.0121283	0.73342017	791
NEUT	Glu	-0.0129143	0.8762033	148
PLT	Glu	-0.0599613	0.47064163	147
RBC	Glu	0.08107149	0.32566235	149
RDW	Glu	0.00401853	0.96133917	148
SHBG	Glu	-0.0367713	0.4421858	439
Tes	Glu	0.02886628	0.43549723	732
Tg	Glu	0.1130901	6.14E-04	914
TIBC	Glu	0.03565067	0.45213024	447
WBC	Glu	-0.0379772	0.28393613	798
Alb	Hb	0.3709026	2.31E-14	448
ALT	Hb	0.04642881	0.52657721	546
AST	Hb	-0.0010143	0.98778824	457
B12	Hb	0.11151035	0.00945748	828
BASOS	Hb	-0.0632436	0.67467859	165
Ca	Hb	0.33116799	0	882
Chol	Hb	0.26344957	6.11E-14	891

CK	Hb	-0.1396075	3.42E-04	897
Cor	Hb	0.04754359	0.54010782	483
D	Hb	0.02425679	0.72226809	893
EOS	Hb	0.014503	0.93219501	165
FE	Hb	0.10760174	0.09409729	450
Fer	Hb	0.07893855	0.07503073	921
Fol	Hb	0.05409403	0.30335824	870
FT	Hb	0.20789849	0.00255401	302
GGT	Hb	0.12340934	0.04336036	458
Glu	Hb	0.05704993	0.26152087	885
Hb	Hb	1	0	939
HCT	Hb	0.54258465	4.31E-14	166
HDL	Hb	0.1065148	0.00160374	875
hsCRP	Hb	-0.0169113	0.6923033	550
K	Hb	0.15665913	1.02E-05	786
LDL	Hb	0.2183762	8.69E-11	864
LYMPHS	Hb	0.18054744	0.02030589	165
MCH	Hb	0.09462776	0.22524302	166
MCHC	Hb	0.04249119	0.58673776	166
MCV	Hb	0.02819498	0.71840323	166
Mg	Hb	0.11198693	9.18E-04	873
MONOS	Hb	0.0891646	0.25474365	165
MPV	Hb	-0.0554144	0.48095703	164
Na	Hb	-0.003934	0.91226069	787
NEUT	Hb	0.18940616	0.01482741	165
PLT	Hb	0.15558007	0.04599526	165
RBC	Hb	0.66887957	0	166
RDW	Hb	0.02931356	0.70773074	166
SHBG	Hb	0.00935291	0.84007496	468
Tes	Hb	0.13282925	2.72E-04	747
Tg	Hb	0.13264094	7.85E-05	881
TIBC	Hb	0.2149454	4.53E-06	447
WBC	Hb	0.16015098	4.05E-06	820
Alb	HCT	0.38262426	1.28E-04	127
ALT	HCT	-0.1792471	0.13227177	140
AST	HCT	-0.1107322	0.45512983	126
B12	HCT	0.04104154	0.80651767	155
BASOS	HCT	-0.0900696	0.48536878	167
Ca	HCT	0.21978851	0.03912429	148
Chol	HCT	0.19770016	0.07503073	147
CK	HCT	-0.2064214	0.04173587	165
Cor	HCT	0.02293889	0.89691237	142
D	HCT	0.01254929	0.93992327	160

EOS	HCT	-0.0336312	0.83938504	167
FE	HCT	-0.0303401	0.86364788	128
Fer	HCT	0.00351333	0.98370723	167
Fol	HCT	-0.1649156	0.16086825	148
FT	HCT	0.06265986	0.78837319	77
GGT	HCT	0.17891767	0.16007966	127
Glu	HCT	0.01691135	0.92547213	148
Hb	HCT	0.54258465	1.77E-12	166
HCT	HCT	1	0	168
HDL	HCT	0.07742328	0.3529601	146
hsCRP	HCT	-0.1553522	0.05517442	153
K	HCT	0.11801149	0.16961647	137
LDL	HCT	0.1616202	0.05050172	147
LYMPHS	HCT	0.1666045	0.03140552	167
MCH	HCT	-0.0933244	0.2288917	168
MCHC	HCT	-0.5554447	5.55E-15	168
MCV	HCT	0.47761176	5.90E-11	168
Mg	HCT	0.04939102	0.54971853	149
MONOS	HCT	-0.1250052	0.10748311	167
MPV	HCT	-2.82E-05	0.99971215	166
Na	HCT	0.11718106	0.17266039	137
NEUT	HCT	0.10981816	0.15772015	167
PLT	HCT	0.17144034	0.02674053	167
RBC	HCT	0.77325741	0	168
RDW	HCT	0.30369589	6.28E-05	168
SHBG	HCT	0.15939618	0.06089118	139
Tes	HCT	0.18768825	0.02016722	153
Tg	HCT	0.24733093	0.00252611	147
TIBC	HCT	0.28415968	0.00115145	128
WBC	HCT	0.13822368	0.08036027	161
Alb	HDL	0.26283558	2.75E-07	458
ALT	HDL	0.04092234	0.60106686	521
AST	HDL	0.11055084	0.07503073	469
B12	HDL	0.09821728	0.03326381	785
BASOS	HDL	-0.0412683	0.81395677	146
Ca	HDL	0.18644854	5.78E-07	880
Chol	HDL	0.40199454	0	937
CK	HDL	0.06710711	0.17564155	852
Cor	HDL	0.06951553	0.34140877	463
D	HDL	0.09501947	0.0303779	865
EOS	HDL	-0.0791174	0.59400332	146
FE	HDL	0.03101076	0.74778537	453
Fer	HDL	0.06779782	0.16007966	880

Fol	HDL	0.05813139	0.25798098	864
FT	HDL	-0.0324342	0.78602851	294
GGT	HDL	0.1063116	0.09137386	468
Glu	HDL	-0.0175806	0.79296579	908
Hb	HDL	0.1065148	0.01116888	875
HCT	HDL	0.07742328	0.60111109	146
HDL	HDL	1	0	938
hsCRP	HDL	-0.0462995	0.28826767	528
K	HDL	0.02799432	0.43231064	789
LDL	HDL	0.15348879	2.95E-06	919
LYMPHS	HDL	-0.0127433	0.87866564	146
MCH	HDL	0.17286404	0.03693292	146
MCHC	HDL	-0.0721323	0.38692146	146
MCV	HDL	0.18162533	0.02823505	146
Mg	HDL	0.04664605	0.17023218	866
MONOS	HDL	-0.0952247	0.25290817	146
MPV	HDL	-0.0200496	0.81147617	144
Na	HDL	0.02807215	0.43044626	791
NEUT	HDL	-0.1455719	0.07957044	146
PLT	HDL	0.04662259	0.57762706	145
RBC	HDL	-0.0718705	0.38865148	146
RDW	HDL	0.0717097	0.38971639	146
SHBG	HDL	-0.018007	0.70641577	440
Tes	HDL	-0.0132086	0.72365529	719
Tg	HDL	-0.163213	5.09E-07	937
TIBC	HDL	0.12983418	0.00586673	449
WBC	HDL	0.03306916	0.351448	796
Alb	hsCRP	-0.0635923	0.39940307	453
ALT	hsCRP	0.14436575	0.00826996	508
AST	hsCRP	0.1590171	0.00555336	453
B12	hsCRP	-0.0268902	0.75494317	557
BASOS	hsCRP	-0.0073897	0.96651855	152
Ca	hsCRP	-0.0149342	0.86364788	523
Chol	hsCRP	-0.026525	0.76360462	533
CK	hsCRP	0.1498081	0.00354394	551
Cor	hsCRP	0.0723816	0.29408306	499
D	hsCRP	0.00973723	0.90893975	568
EOS	hsCRP	0.17477549	0.12371871	152
FE	hsCRP	-0.0759481	0.29408306	454
Fer	hsCRP	0.01137315	0.89691237	562
Fol	hsCRP	-0.0270624	0.76360462	521
FT	hsCRP	-0.0586968	0.55316443	303
GGT	hsCRP	0.17468795	0.00183535	454

Glu	hsCRP	-0.0458841	0.53604032	536
Hb	hsCRP	-0.0169113	0.85172962	550
HCT	hsCRP	-0.1553522	0.18391473	153
HDL	hsCRP	-0.0462995	0.53604032	528
hsCRP	hsCRP	1	0	590
K	hsCRP	0.0575381	0.19278574	514
LDL	hsCRP	-0.0289773	0.50886594	522
LYMPHS	hsCRP	-0.0130141	0.87356576	152
MCH	hsCRP	0.05429305	0.5050581	153
MCHC	hsCRP	0.11973224	0.14043608	153
MCV	hsCRP	-0.1195826	0.14093638	153
Mg	hsCRP	0.07979666	0.06823868	523
MONOS	hsCRP	0.19364909	0.01682862	152
MPV	hsCRP	-0.0465006	0.57073636	151
Na	hsCRP	-0.0074006	0.86720195	513
NEUT	hsCRP	0.18040426	0.02614214	152
PLT	hsCRP	0.00186839	0.98177401	152
RBC	hsCRP	-0.0846234	0.29673256	154
RDW	hsCRP	-0.114964	0.15705067	153
SHBG	hsCRP	-0.0872985	0.05833394	471
Tes	hsCRP	-0.0641384	0.12987358	559
Tg	hsCRP	0.10801759	0.01267129	532
TIBC	hsCRP	0.0773785	0.10114265	450
WBC	hsCRP	0.21982809	3.36E-07	528
Alb	K	0.04872429	0.54444952	452
ALT	K	0.14031939	0.01022229	513
AST	K	0.15974945	0.00476229	462
B12	K	0.06374085	0.2345625	777
BASOS	K	0.04205769	0.8159548	137
Ca	K	0.22168877	9.31E-09	790
Chol	K	0.08261282	0.08672671	794
CK	K	0.04767825	0.40932407	763
Cor	K	0.11156448	0.0776948	453
D	K	0.02301087	0.75494317	760
EOS	K	-0.0476321	0.78602851	137
FE	K	0.02164067	0.83281258	453
Fer	K	0.00673073	0.93069407	784
Fol	K	0.03570038	0.57261873	773
FT	K	0.06108679	0.54444952	288
GGT	K	-0.011076	0.90725634	462
Glu	K	0.10071487	0.02814996	792
Hb	K	0.15665913	1.42E-04	786
HCT	K	0.11801149	0.38912013	137

HDL	K	0.02799432	0.68787578	789
hsCRP	K	0.0575381	0.41425035	514
K	K	1	0	802
LDL	K	0.0981472	0.00614763	778
LYMPHS	K	-0.0278572	0.74659251	137
MCH	K	-0.0505535	0.55742711	137
MCHC	K	0.11445469	0.18293776	137
MCV	K	-0.1149591	0.18100323	137
Mg	K	0.07878214	0.02809849	777
MONOS	K	0.0743196	0.38807558	137
MPV	K	0.0039617	0.96362671	135
Na	K	0.14124092	6.37E-05	796
NEUT	K	0.21173043	0.01300031	137
PLT	K	0.16764196	0.05107991	136
RBC	K	0.23427013	0.00586222	137
RDW	K	-0.115795	0.17783047	137
SHBG	K	0.02091957	0.66275751	437
Tes	K	0.02243262	0.55095107	709
Tg	K	-0.0125372	0.72429069	794
TIBC	K	-0.0191783	0.68526766	449
WBC	K	-0.0456663	0.20265968	780
Alb	LDL	0.33829342	6.42E-12	450
ALT	LDL	-0.0284751	0.75208683	514
AST	LDL	-0.0508772	0.52315013	461
B12	LDL	0.0521494	0.3576892	774
BASOS	LDL	0.04826884	0.77675523	147
Ca	LDL	0.2136836	6.56E-09	868
Chol	LDL	0.83601357	0	927
CK	LDL	-0.0426948	0.45498275	840
Cor	LDL	0.06990083	0.34140877	457
D	LDL	-0.0494735	0.35820013	857
EOS	LDL	0.12758167	0.32835866	147
FE	LDL	0.04509178	0.59400332	445
Fer	LDL	0.16110693	3.04E-05	868
Fol	LDL	0.03047544	0.62920989	852
FT	LDL	-0.0632785	0.53442477	286
GGT	LDL	0.10716775	0.09137386	460
Glu	LDL	0.04453519	0.40496377	896
Hb	LDL	0.2183762	2.95E-09	864
HCT	LDL	0.1616202	0.17571086	147
HDL	LDL	0.15348879	4.79E-05	919
hsCRP	LDL	-0.0289773	0.74778537	522
K	LDL	0.0981472	0.0342511	778

LDL	LDL	1	0	928
LYMPHS	LDL	0.15976564	0.05324187	147
MCH	LDL	-0.029058	0.72680765	147
MCHC	LDL	0.08912322	0.28305813	147
MCV	LDL	-0.1285973	0.12059008	147
Mg	LDL	0.02722681	0.42682375	854
MONOS	LDL	0.02776315	0.73852966	147
MPV	LDL	-0.0569026	0.49661911	145
Na	LDL	-0.0418816	0.24267498	780
NEUT	LDL	0.02634401	0.75144665	147
PLT	LDL	0.07431555	0.37267534	146
RBC	LDL	0.24141933	0.00322156	147
RDW	LDL	-0.0872194	0.29350898	147
SHBG	LDL	0.0113005	0.81482712	432
Tes	LDL	5.35E-04	0.98866288	707
Tg	LDL	0.18191357	2.44E-08	927
TIBC	LDL	0.16104326	6.88E-04	441
WBC	LDL	0.06100517	0.08761756	785
Alb	LYMPHS	0.10767004	0.46605252	127
ALT	LYMPHS	0.05256727	0.76360462	139
AST	LYMPHS	-0.1022329	0.4882065	126
B12	LYMPHS	0.14168121	0.24172619	154
BASOS	LYMPHS	-0.0015647	0.98778824	167
Ca	LYMPHS	0.04738032	0.78059165	148
Chol	LYMPHS	0.12292917	0.34385462	147
CK	LYMPHS	-0.0294848	0.85512048	164
Cor	LYMPHS	0.13743269	0.29009771	141
D	LYMPHS	-0.0253185	0.87261541	159
EOS	LYMPHS	0.18283091	0.07947184	167
FE	LYMPHS	0.02208648	0.90564582	128
Fer	LYMPHS	0.33245104	1.65E-04	166
Fol	LYMPHS	-0.1140474	0.38622319	148
FT	LYMPHS	0.02355656	0.92547213	77
GGT	LYMPHS	0.03248161	0.86212334	127
Glu	LYMPHS	-0.0586264	0.72419059	148
Hb	LYMPHS	0.18054744	0.08750603	165
HCT	LYMPHS	0.1666045	0.12371871	167
HDL	LYMPHS	-0.0127433	0.94051362	146
hsCRP	LYMPHS	-0.0130141	0.93983626	152
K	LYMPHS	-0.0278572	0.8704666	137
LDL	LYMPHS	0.15976564	0.18134784	147
LYMPHS	LYMPHS	1	0	167
MCH	LYMPHS	-0.0996476	0.20010602	167

MCHC	LYMPHS	-0.0669025	0.39031841	167
MCV	LYMPHS	-0.0328131	0.67377915	167
Mg	LYMPHS	0.09505831	0.24883823	149
MONOS	LYMPHS	0.28205938	2.22E-04	167
MPV	LYMPHS	-0.1369458	0.07943046	165
Na	LYMPHS	0.14294753	0.09563443	137
NEUT	LYMPHS	0.09058652	0.24432732	167
PLT	LYMPHS	0.1426937	0.06665637	166
RBC	LYMPHS	0.15566193	0.04456386	167
RDW	LYMPHS	0.02686995	0.73032711	167
SHBG	LYMPHS	0.0306851	0.72088698	138
Tes	LYMPHS	-0.0017994	0.98244642	152
Tg	LYMPHS	0.01338404	0.87217631	147
TIBC	LYMPHS	0.00728907	0.93491831	128
WBC	LYMPHS	0.33778242	1.25E-05	160
Alb	MCH	-0.0842207	0.59611788	127
ALT	MCH	0.07187977	0.65058464	140
AST	MCH	0.00887292	0.96644795	126
B12	MCH	0.1359122	0.26567463	155
BASOS	MCH	0.05521167	0.72419059	167
Ca	MCH	0.08349052	0.56003183	148
Chol	MCH	-0.016246	0.92848065	147
CK	MCH	0.02914108	0.85621892	165
Cor	MCH	-0.0589288	0.72766479	142
D	MCH	0.04884412	0.76360462	160
EOS	MCH	0.01996136	0.90330071	167
FE	MCH	-0.2202697	0.06082069	128
Fer	MCH	-0.117059	0.33685219	167
Fol	MCH	0.09429745	0.4882065	148
FT	MCH	0.0363725	0.87314606	77
GGT	MCH	-0.0582573	0.75129675	127
Glu	MCH	-0.037468	0.83540593	148
Hb	MCH	0.09462776	0.46602004	166
HCT	MCH	-0.0933244	0.46615019	168
HDL	MCH	0.17286404	0.14121412	146
hsCRP	MCH	0.05429305	0.74610856	153
K	MCH	-0.0505535	0.77641633	137
LDL	MCH	-0.029058	0.86364788	147
LYMPHS	MCH	-0.0996476	0.42529346	167
MCH	MCH	1	0	168
MCHC	MCH	0.36526364	1.13E-06	168
MCV	MCH	0.25903507	6.98E-04	168
Mg	MCH	0.09581507	0.2450736	149

MONOS	MCH	0.00333884	0.96584236	167
MPV	MCH	0.27827642	2.83E-04	166
Na	MCH	0.0464096	0.59021567	137
NEUT	MCH	-0.234166	0.00232043	167
PLT	MCH	-0.0895595	0.24973829	167
RBC	MCH	-0.2790969	2.49E-04	168
RDW	MCH	-0.17168	0.0260704	168
SHBG	MCH	0.06080352	0.47705467	139
Tes	MCH	-0.0369811	0.64994802	153
Tg	MCH	-0.2256742	0.00598971	147
TIBC	MCH	-0.0401322	0.65287525	128
WBC	MCH	-0.1606951	0.04171438	161
Alb	MCHC	-0.0646167	0.72226809	127
ALT	MCHC	0.17838949	0.13435131	140
AST	MCHC	0.02127918	0.90725634	126
B12	MCHC	0.0276511	0.86364788	155
BASOS	MCHC	0.08303743	0.53442477	167
Ca	MCHC	0.11091148	0.40254267	148
Chol	MCHC	0.02998694	0.86212334	147
CK	MCHC	-0.0110667	0.94473419	165
Cor	MCHC	0.02201965	0.90106041	142
D	MCHC	0.03961535	0.81273836	160
EOS	MCHC	0.01484006	0.93012599	167
FE	MCHC	0.108563	0.46164558	128
Fer	MCHC	0.0550188	0.72422101	167
Fol	MCHC	0.20273935	0.06403908	148
FT	MCHC	0.173018	0.33685219	77
GGT	MCHC	-0.0355355	0.85172962	127
Glu	MCHC	0.05301879	0.75208683	148
Hb	MCHC	0.04249119	0.78770302	166
HCT	MCHC	-0.5554447	2.41E-13	168
HDL	MCHC	-0.0721323	0.64349411	146
hsCRP	MCHC	0.11973224	0.34678373	153
K	MCHC	0.11445469	0.40496377	137
LDL	MCHC	0.08912322	0.53329792	147
LYMPHS	MCHC	-0.0669025	0.64365404	167
MCH	MCHC	0.36526364	1.95E-05	168
MCHC	MCHC	1	0	168
MCV	MCHC	-0.7238269	0	168
Mg	MCHC	0.03963556	0.63127879	149
MONOS	MCHC	0.23882588	0.00188209	167
MPV	MCHC	0.02641839	0.7354656	166
Na	MCHC	-0.1463222	0.08797813	137

NEUT	MCHC	0.04809838	0.5370653	167
PLT	MCHC	0.06455988	0.4071631	167
RBC	MCHC	-0.1050156	0.17549925	168
RDW	MCHC	-0.5682042	8.88E-16	168
SHBG	MCHC	-0.1739013	0.04062085	139
Tes	MCHC	-0.0106666	0.89588569	153
Tg	MCHC	-0.0815136	0.3263473	147
TIBC	MCHC	-0.07495	0.40044445	128
WBC	MCHC	-0.0342993	0.66578141	161
Alb	MCV	0.04215038	0.82799561	127
ALT	MCV	-0.1283161	0.33685219	140
AST	MCV	-0.0019118	0.98778824	126
B12	MCV	0.02320959	0.88694962	155
BASOS	MCV	-0.0342942	0.83837585	167
Ca	MCV	-0.0313786	0.85512048	148
Chol	MCV	-0.0564085	0.73896323	147
CK	MCV	0.02480699	0.87261541	165
Cor	MCV	-0.0558828	0.74778537	142
D	MCV	-0.0143935	0.93321673	160
EOS	MCV	-0.0353425	0.83540593	167
FE	MCV	-0.181632	0.15064969	128
Fer	MCV	-0.079399	0.55316443	167
Fol	MCV	-0.1645688	0.16106994	148
FT	MCV	-0.1503671	0.41425035	77
GGT	MCV	-0.0073963	0.96715687	127
Glu	MCV	-0.0589096	0.72419059	148
Hb	MCV	0.02819498	0.86212334	166
HCT	MCV	0.47761176	2.09E-09	168
HDL	MCV	0.18162533	0.11352235	146
hsCRP	MCV	-0.1195826	0.34678373	153
K	MCV	-0.1149591	0.40453444	137
LDL	MCV	-0.1285973	0.32323114	147
LYMPHS	MCV	-0.0328131	0.8434526	167
MCH	MCV	0.25903507	0.00555336	168
MCHC	MCV	-0.7238269	0	168
MCV	MCV	1	0	168
Mg	MCV	0.00659268	0.93639873	149
MONOS	MCV	-0.2524903	9.95E-04	167
MPV	MCV	0.14156335	0.06886456	166
Na	MCV	0.17701763	0.03851558	137
NEUT	MCV	-0.1958824	0.01118158	167
PLT	MCV	-0.1483276	0.05574624	167
RBC	MCV	-0.0815599	0.29326018	168

RDW	MCV	0.44635243	1.33E-09	168
SHBG	MCV	0.17322547	0.04142015	139
Tes	MCV	-0.0123902	0.87918099	153
Tg	MCV	-0.0589067	0.47849564	147
TIBC	MCV	0.05177289	0.56165378	128
WBC	MCV	-0.0696339	0.38008671	161
Alb	Mg	0.08808234	0.19550825	457
ALT	Mg	0.09574817	0.12371871	507
AST	Mg	0.15112282	0.00889319	456
B12	Mg	0.05235443	0.34529923	800
BASOS	Mg	-0.1184035	0.36202563	149
Ca	Mg	0.13722925	4.92E-04	886
Chol	Mg	0.03297565	0.57877658	882
CK	Mg	0.09340974	0.03326381	871
Cor	Mg	0.0537739	0.4865217	461
D	Mg	-0.0134304	0.85294133	857
EOS	Mg	0.02192691	0.89771341	149
FE	Mg	0.03470431	0.71062566	459
Fer	Mg	0.02202663	0.74909171	885
Fol	Mg	0.06260033	0.20393945	881
FT	Mg	-0.0271834	0.83258014	292
GGT	Mg	0.02092855	0.83762863	457
Glu	Mg	-0.0314725	0.60106686	876
Hb	Mg	0.11198693	0.00716044	873
HCT	Mg	0.04939102	0.77014666	149
HDL	Mg	0.04664605	0.38938739	866
hsCRP	Mg	0.07979666	0.21462165	523
K	Mg	0.07878214	0.11352235	777
LDL	Mg	0.02722681	0.68221828	854
LYMPHS	Mg	0.09505831	0.4865217	149
MCH	Mg	0.09581507	0.48418067	149
MCHC	Mg	0.03963556	0.82066243	149
MCV	Mg	0.00659268	0.96740531	149
Mg	Mg	1	0	890
MONOS	Mg	0.01427197	0.86284547	149
MPV	Mg	-0.0068122	0.93473486	147
Na	Mg	0.04723744	0.18839403	777
NEUT	Mg	-0.0209376	0.79991914	149
PLT	Mg	0.15040748	0.06805149	148
RBC	Mg	0.09920763	0.22710183	150
RDW	Mg	0.05332002	0.51838816	149
SHBG	Mg	-0.0427905	0.36891736	443
Tes	Mg	-0.0538293	0.15161494	711

Tg	Mg	-0.0082867	0.80695365	872
TIBC	Mg	0.14568938	0.00183476	455
WBC	Mg	-0.038317	0.28639686	776
Alb	MONOS	0.03786668	0.8433749	127
ALT	MONOS	0.14899808	0.24191374	139
AST	MONOS	0.01047472	0.95896427	126
B12	MONOS	-0.0980671	0.46605252	154
BASOS	MONOS	0.21836086	0.02814996	167
Ca	MONOS	0.04267961	0.80183542	148
Chol	MONOS	-0.0046307	0.98075643	147
CK	MONOS	-0.0541398	0.73385631	164
Cor	MONOS	0.08882098	0.54000743	141
D	MONOS	-0.0391287	0.81573963	159
EOS	MONOS	0.14998683	0.18134784	167
FE	MONOS	-0.1707902	0.18205756	128
Fer	MONOS	0.12036359	0.32702255	166
Fol	MONOS	-0.0097738	0.95896427	148
FT	MONOS	0.12860354	0.50660241	77
GGT	MONOS	-0.0022198	0.98778824	127
Glu	MONOS	-0.1073045	0.4162572	148
Hb	MONOS	0.0891646	0.4882065	165
HCT	MONOS	-0.1250052	0.29624321	167
HDL	MONOS	-0.0952247	0.4882065	146
hsCRP	MONOS	0.19364909	0.07543864	152
K	MONOS	0.0743196	0.64362666	137
LDL	MONOS	0.02776315	0.86364788	147
LYMPHS	MONOS	0.28205938	0.00216167	167
MCH	MONOS	0.00333884	0.98370723	167
MCHC	MONOS	0.23882588	0.01265541	167
MCV	MONOS	-0.2524903	0.00753621	167
Mg	MONOS	0.01427197	0.9369507	149
MONOS	MONOS	1	0	167
MPV	MONOS	0.07443156	0.34204062	165
Na	MONOS	-0.0258438	0.76435112	137
NEUT	MONOS	0.38658234	2.47E-07	167
PLT	MONOS	0.156004	0.04474021	166
RBC	MONOS	0.03414092	0.66137771	167
RDW	MONOS	-0.3387033	7.56E-06	167
SHBG	MONOS	0.01215891	0.88744271	138
Tes	MONOS	0.05867694	0.47271711	152
Tg	MONOS	0.14097928	0.0885291	147
TIBC	MONOS	0.10223969	0.25081376	128
WBC	MONOS	0.42060522	3.07E-08	160

Alb	MPV	0.05194453	0.78011122	125
ALT	MPV	0.02881698	0.86364788	138
AST	MPV	0.16190397	0.22503508	124
B12	MPV	-0.0944472	0.48418067	153
BASOS	MPV	0.25405099	0.00753621	165
Ca	MPV	0.08765905	0.5386753	146
Chol	MPV	-0.1217099	0.35393755	145
CK	MPV	0.07467022	0.59404482	163
Cor	MPV	0.03287637	0.85465188	140
D	MPV	0.12077038	0.33685219	158
EOS	MPV	0.04024733	0.80212855	165
FE	MPV	-0.1352883	0.33685219	126
Fer	MPV	-0.1493286	0.18424605	165
Fol	MPV	0.05094975	0.76360462	146
FT	MPV	0.04363259	0.85512048	76
GGT	MPV	-0.1334562	0.34385462	125
Glu	MPV	0.10191854	0.46073756	146
Hb	MPV	-0.0554144	0.72422101	164
HCT	MPV	-2.82E-05	0.99971215	166
HDL	MPV	-0.0200496	0.90725634	144
hsCRP	MPV	-0.0465006	0.78100765	151
K	MPV	0.0039617	0.98370723	135
LDL	MPV	-0.0569026	0.73896323	145
LYMPHS	MPV	-0.1369458	0.24172619	165
MCH	MPV	0.27827642	0.00259858	166
MCHC	MPV	0.02641839	0.86364788	166
MCV	MPV	0.14156335	0.21572031	166
Mg	MPV	-0.0068122	0.96715687	147
MONOS	MPV	0.07443156	0.59400332	165
MPV	MPV	1	0	166
Na	MPV	0.01108984	0.89841959	135
NEUT	MPV	-0.1417661	0.06931531	165
PLT	MPV	-0.1936628	0.01241824	166
RBC	MPV	-0.1095271	0.16010303	166
RDW	MPV	-0.3427727	6.16E-06	166
SHBG	MPV	0.04632611	0.59088521	137
Tes	MPV	0.01738951	0.83216462	151
Tg	MPV	-0.1524039	0.06724887	145
TIBC	MPV	0.16993883	0.05711768	126
WBC	MPV	-0.1179906	0.13728724	160
Alb	Na	0.10412383	0.10910562	452
ALT	Na	0.00386417	0.96651855	514
AST	Na	0.01354278	0.88606763	463

B12	Na	0.05672509	0.30889742	779
BASOS	Na	0.05746944	0.74610856	137
Ca	Na	0.13872451	9.75E-04	789
Chol	Na	-0.0552583	0.32084304	796
CK	Na	0.01987391	0.78727224	764
Cor	Na	0.00854723	0.93321673	453
D	Na	-0.0060926	0.93752823	761
EOS	Na	0.02130276	0.90564582	137
FE	Na	-0.0603926	0.42514373	453
Fer	Na	0.00339583	0.96644795	785
Fol	Na	-0.045654	0.43172609	775
FT	Na	-0.0830984	0.37277721	288
GGT	Na	0.01276297	0.89691237	462
Glu	Na	-0.0121283	0.86364788	791
Hb	Na	-0.003934	0.96179801	787
HCT	Na	0.11718106	0.39378685	137
HDL	Na	0.02807215	0.68660139	791
hsCRP	Na	-0.0074006	0.93752823	513
K	Na	0.14124092	7.00E-04	796
LDL	Na	-0.0418816	0.48413956	780
LYMPHS	Na	0.14294753	0.27525778	137
MCH	Na	0.0464096	0.78919601	137
MCHC	Na	-0.1463222	0.25798098	137
MCV	Na	0.17701763	0.14557849	137
Mg	Na	0.04723744	0.40932407	777
MONOS	Na	-0.0258438	0.88064088	137
MPV	Na	0.01108984	0.95342487	135
Na	Na	1	0	803
NEUT	Na	0.04694085	0.58596346	137
PLT	Na	-0.0185178	0.83056403	136
RBC	Na	0.01421859	0.86901655	137
RDW	Na	0.12069482	0.16005281	137
SHBG	Na	4.47E-04	0.99256355	437
Tes	Na	-0.0166822	0.65721631	710
Tg	Na	0.02415823	0.49611458	796
TIBC	Na	-0.0806394	0.08787126	449
WBC	Na	-0.0588366	0.10015227	782
Alb	NEUT	0.21245002	0.07503073	127
ALT	NEUT	0.11353245	0.40496377	139
AST	NEUT	-0.0860206	0.59146704	126
B12	NEUT	-0.1238001	0.33227201	154
BASOS	NEUT	0.10232814	0.40932407	167
Ca	NEUT	0.17086528	0.14406389	148

Chol	NEUT	0.05608634	0.74121188	147
CK	NEUT	-0.1509033	0.18205756	164
Cor	NEUT	-0.0546268	0.75208683	141
D	NEUT	-0.2046092	0.04934308	159
EOS	NEUT	0.00331844	0.98370723	167
FE	NEUT	0.02636955	0.8831266	128
Fer	NEUT	0.02094435	0.89691237	166
Fol	NEUT	-0.1240124	0.33726913	148
FT	NEUT	0.07604173	0.74778537	77
GGT	NEUT	0.11637529	0.41425035	127
Glu	NEUT	-0.0129143	0.94008057	148
Hb	NEUT	0.18940616	0.06967097	165
HCT	NEUT	0.10981816	0.37166681	167
HDL	NEUT	-0.1455719	0.24172619	146
hsCRP	NEUT	0.18040426	0.10732038	152
K	NEUT	0.21173043	0.06221005	137
LDL	NEUT	0.02634401	0.87261541	147
LYMPHS	NEUT	0.09058652	0.48418067	167
MCH	NEUT	-0.234166	0.0153384	167
MCHC	NEUT	0.04809838	0.76360462	167
MCV	NEUT	-0.1958824	0.05590787	167
Mg	NEUT	-0.0209376	0.90425642	149
MONOS	NEUT	0.38658234	4.69E-06	167
MPV	NEUT	-0.1417661	0.21626377	165
Na	NEUT	0.04694085	0.78770302	137
NEUT	NEUT	1	0	167
PLT	NEUT	0.30843069	5.28E-05	166
RBC	NEUT	0.30587639	5.82E-05	167
RDW	NEUT	-0.0852286	0.273465	167
SHBG	NEUT	0.06539227	0.44605165	138
Tes	NEUT	-0.0774862	0.34269422	152
Tg	NEUT	0.3889513	1.12E-06	147
TIBC	NEUT	0.10592976	0.23402544	128
WBC	NEUT	0.7233403	0	160
Alb	PLT	0.16792597	0.19555142	126
ALT	PLT	0.14634321	0.25559933	139
AST	PLT	-0.0029188	0.98778824	125
B12	PLT	0.09614162	0.47480583	154
BASOS	PLT	-0.0467053	0.77014666	166
Ca	PLT	0.07604753	0.61033013	147
Chol	PLT	0.10437601	0.44254806	146
CK	PLT	-0.0783041	0.56927105	164
Cor	PLT	0.22014309	0.04531675	141

D	PLT	-0.130057	0.28901331	159
EOS	PLT	-0.0661979	0.64882265	166
FE	PLT	-0.0433151	0.81869301	127
Fer	PLT	0.03340738	0.84050023	166
Fol	PLT	-0.1254108	0.33685219	147
FT	PLT	0.15219492	0.40931471	77
GGT	PLT	0.20400368	0.09253792	126
Glu	PLT	-0.0599613	0.72226809	147
Hb	PLT	0.15558007	0.16160498	165
HCT	PLT	0.17144034	0.10910562	167
HDL	PLT	0.04662259	0.7851637	145
hsCRP	PLT	0.00186839	0.98778824	152
K	PLT	0.16764196	0.17629349	136
LDL	PLT	0.07431555	0.62783319	146
LYMPHS	PLT	0.1426937	0.21308183	166
MCH	PLT	-0.0895595	0.4865217	167
MCHC	PLT	0.06455988	0.66164004	167
MCV	PLT	-0.1483276	0.18424605	167
Mg	PLT	0.15040748	0.21462165	148
MONOS	PLT	0.156004	0.16007966	166
MPV	PLT	-0.1936628	0.06082069	166
Na	PLT	-0.0185178	0.92153619	136
NEUT	PLT	0.30843069	6.06E-04	166
PLT	PLT	1	0	167
RBC	PLT	0.31053015	4.42E-05	167
RDW	PLT	-0.093846	0.22769687	167
SHBG	PLT	0.12421711	0.14661959	138
Tes	PLT	0.0938503	0.25012205	152
Tg	PLT	0.21302266	0.00983609	146
TIBC	PLT	0.17791567	0.0453731	127
WBC	PLT	0.15913381	0.04443698	160
Alb	RBC	0.44905935	2.17E-06	128
ALT	RBC	-0.0445881	0.79711481	140
AST	RBC	-0.0987022	0.51412351	127
B12	RBC	0.02786683	0.86364788	155
BASOS	RBC	-0.0761636	0.57877658	167
Ca	RBC	0.31112178	0.00116943	149
Chol	RBC	0.2313729	0.02885667	147
CK	RBC	-0.2782369	0.00265593	165
Cor	RBC	0.07149314	0.64882265	143
D	RBC	-0.0316337	0.85172962	161
EOS	RBC	-0.0513739	0.74778537	167
FE	RBC	0.13620861	0.32835866	129

Fer	RBC	0.09259527	0.47117049	168
Fol	RBC	-0.033354	0.85111048	149
FT	RBC	0.24480807	0.12498792	77
GGT	RBC	0.22863041	0.04843294	128
Glu	RBC	0.08107149	0.57852475	149
Hb	RBC	0.66887957	0	166
HCT	RBC	0.77325741	0	168
HDL	RBC	-0.0718705	0.64362666	146
hsCRP	RBC	-0.0846234	0.54010782	154
K	RBC	0.23427013	0.03326381	137
LDL	RBC	0.24141933	0.02087621	147
LYMPHS	RBC	0.15566193	0.16007966	167
MCH	RBC	-0.2790969	0.00239509	168
MCHC	RBC	-0.1050156	0.39793433	168
MCV	RBC	-0.0815599	0.5386753	168
Mg	RBC	0.09920763	0.46605252	150
MONOS	RBC	0.03414092	0.83882051	167
MPV	RBC	-0.1095271	0.37277721	166
Na	RBC	0.01421859	0.93752823	137
NEUT	RBC	0.30587639	6.58E-04	167
PLT	RBC	0.31053015	5.15E-04	167
RBC	RBC	1	0	169
RDW	RBC	0.00354133	0.96366232	168
SHBG	RBC	0.0484073	0.57006153	140
Tes	RBC	0.26443	9.57E-04	153
Tg	RBC	0.34707795	1.66E-05	147
TIBC	RBC	0.32454549	1.75E-04	129
WBC	RBC	0.24738796	0.00155686	161
Alb	RDW	-0.0090025	0.96644795	127
ALT	RDW	-0.2049043	0.07038261	140
AST	RDW	-0.1261119	0.37277721	126
B12	RDW	-0.0320749	0.85172962	155
BASOS	RDW	-0.3174217	3.61E-04	167
Ca	RDW	-0.1167766	0.37166681	148
Chol	RDW	0.00914369	0.96179801	147
CK	RDW	0.06055933	0.69483209	165
Cor	RDW	-0.0663099	0.68787578	142
D	RDW	-0.0475172	0.77014666	160
EOS	RDW	-0.0252723	0.8704666	167
FE	RDW	-0.0134501	0.94051362	128
Fer	RDW	-0.128924	0.27763476	167
Fol	RDW	-0.207764	0.05605099	148
FT	RDW	-0.1635241	0.37045732	77

GGT	RDW	-0.0131788	0.94228326	127
Glu	RDW	0.00401853	0.98370723	148
Hb	RDW	0.02931356	0.85512048	166
HCT	RDW	0.30369589	7.00E-04	168
HDL	RDW	0.0717097	0.64365404	146
hsCRP	RDW	-0.114964	0.37166681	153
K	RDW	-0.115795	0.39973421	137
LDL	RDW	-0.0872194	0.5386753	147
LYMPHS	RDW	0.02686995	0.86364788	167
MCH	RDW	-0.17168	0.10732038	168
MCHC	RDW	-0.5682042	4.33E-14	168
MCV	RDW	0.44635243	3.83E-08	168
Mg	RDW	0.05332002	0.75208683	149
MONOS	RDW	-0.3387033	1.11E-04	167
MPV	RDW	-0.3427727	9.43E-05	166
Na	RDW	0.12069482	0.37277721	137
NEUT	RDW	-0.0852286	0.52025049	167
PLT	RDW	-0.093846	0.46605252	167
RBC	RDW	0.00354133	0.98370723	168
RDW	RDW	1	0	168
SHBG	RDW	0.23718441	0.00493351	139
Tes	RDW	0.06606113	0.41718387	153
Tg	RDW	0.04547835	0.58439824	147
TIBC	RDW	0.01875409	0.83357764	128
WBC	RDW	-0.0194606	0.80643603	161
Alb	SHBG	0.06973117	0.35393755	439
ALT	SHBG	0.03732355	0.70133787	411
AST	SHBG	0.05068419	0.53604032	439
B12	SHBG	0.03713473	0.67467859	473
BASOS	SHBG	-0.1741979	0.15161173	138
Ca	SHBG	0.05764638	0.46605252	442
Chol	SHBG	0.00467744	0.96644795	443
CK	SHBG	0.04496233	0.58465139	466
Cor	SHBG	-0.008086	0.93624215	474
D	SHBG	-0.0761231	0.29009771	459
EOS	SHBG	0.11181195	0.41425035	138
FE	SHBG	-0.0676172	0.37166681	439
Fer	SHBG	0.10203479	0.10732038	476
Fol	SHBG	0.04644216	0.5819754	441
FT	SHBG	-0.1613828	0.02877572	305
GGT	SHBG	-0.0573195	0.46646122	441
Glu	SHBG	-0.0367713	0.69529147	439
Hb	SHBG	0.00935291	0.92550631	468

HCT	SHBG	0.15939618	0.19707519	139
HDL	SHBG	-0.018007	0.85512048	440
hsCRP	SHBG	-0.0872985	0.19117845	471
K	SHBG	0.02091957	0.83920594	437
LDL	SHBG	0.0113005	0.90795022	432
LYMPHS	SHBG	0.0306851	0.86364788	138
MCH	SHBG	0.06080352	0.72419059	139
MCHC	SHBG	-0.1739013	0.15087745	139
MCV	SHBG	0.17322547	0.15239488	139
Mg	SHBG	-0.0427905	0.6228475	443
MONOS	SHBG	0.01215891	0.94473419	138
MPV	SHBG	0.04632611	0.78919601	137
Na	SHBG	4.47E-04	0.9938377	437
NEUT	SHBG	0.06539227	0.69723505	138
PLT	SHBG	0.12421711	0.35738526	138
RBC	SHBG	0.0484073	0.78100765	140
RDW	SHBG	0.23718441	0.02937508	139
SHBG	SHBG	1	0	477
Tes	SHBG	0.08870604	0.05515478	468
Tg	SHBG	0.03048941	0.52260392	442
TIBC	SHBG	0.02933348	0.54081682	437
WBC	SHBG	-0.023598	0.61719945	451
Alb	Tes	-0.0721317	0.33456938	448
ALT	Tes	-0.0613425	0.38622319	507
AST	Tes	-0.0753876	0.29741132	455
B12	Tes	-0.1169822	0.00959059	749
BASOS	Tes	-0.0078413	0.96644795	152
Ca	Tes	0.07673987	0.15064969	714
Chol	Tes	0.00328544	0.96651855	724
CK	Tes	-0.0954974	0.04843294	738
Cor	Tes	0.04746105	0.5422041	481
D	Tes	0.01429724	0.85294133	752
EOS	Tes	-0.0275671	0.86364788	152
FE	Tes	0.02109968	0.83762863	446
Fer	Tes	-0.1136827	0.01153597	762
Fol	Tes	0.03437352	0.61096944	708
FT	Tes	0.66313743	0	304
GGT	Tes	-0.0761112	0.29009771	458
Glu	Tes	0.02886628	0.69042243	732
Hb	Tes	0.13282925	0.00255401	747
HCT	Tes	0.18768825	0.08739128	153
HDL	Tes	-0.0132086	0.86364788	719
hsCRP	Tes	-0.0641384	0.33685219	559

K	Tes	0.02243262	0.77014666	709
LDL	Tes	5.35E-04	0.99120443	707
LYMPHS	Tes	-0.0017994	0.98778824	152
MCH	Tes	-0.0369811	0.83540593	153
MCHC	Tes	-0.0106666	0.95203111	153
MCV	Tes	-0.0123902	0.94051362	153
Mg	Tes	-0.0538293	0.36387585	711
MONOS	Tes	0.05867694	0.72226809	152
MPV	Tes	0.01738951	0.92200058	151
Na	Tes	-0.0166822	0.83762863	710
NEUT	Tes	-0.0774862	0.59400332	152
PLT	Tes	0.0938503	0.4865217	152
RBC	Tes	0.26443	0.00738758	153
RDW	Tes	0.06606113	0.67467859	153
SHBG	Tes	0.08870604	0.18391473	468
Tes	Tes	1	0	782
Tg	Tes	0.01942802	0.60199149	723
TIBC	Tes	0.07506726	0.11421325	444
WBC	Tes	-0.0266277	0.47315947	728
Alb	Tg	0.07866164	0.26567463	460
ALT	Tg	0.11754428	0.0385821	523
AST	Tg	-0.1121134	0.06967801	471
B12	Tg	-0.0535563	0.33685219	790
BASOS	Tg	-0.0483722	0.77675523	147
Ca	Tg	0.10255099	0.01494118	886
Chol	Tg	0.30602501	0	945
CK	Tg	-0.1855939	9.42E-07	858
Cor	Tg	0.08988473	0.17950313	467
D	Tg	-0.1224613	0.00262382	872
EOS	Tg	0.08725867	0.5386753	147
FE	Tg	0.01165145	0.90564582	455
Fer	Tg	0.02587846	0.69529147	886
Fol	Tg	-0.0791056	0.08596426	870
FT	Tg	-0.001228	0.98778824	294
GGT	Tg	0.22060868	2.32E-05	470
Glu	Tg	0.1130901	0.00509582	914
Hb	Tg	0.13264094	8.39E-04	881
HCT	Tg	0.24733093	0.01655771	147
HDL	Tg	-0.163213	9.22E-06	937
hsCRP	Tg	0.10801759	0.06100992	532
K	Tg	-0.0125372	0.86364788	794
LDL	Tg	0.18191357	5.76E-07	927
LYMPHS	Tg	0.01338404	0.93963746	147

MCH	Tg	-0.2256742	0.03361135	147
MCHC	Tg	-0.0815136	0.57852475	147
MCV	Tg	-0.0589067	0.72419059	147
Mg	Tg	-0.0082867	0.90564582	872
MONOS	Tg	0.14097928	0.25862434	147
MPV	Tg	-0.1524039	0.21340979	145
Na	Tg	0.02415823	0.73896323	796
NEUT	Tg	0.3889513	1.95E-05	147
PLT	Tg	0.21302266	0.04981914	146
RBC	Tg	0.34707795	2.19E-04	147
RDW	Tg	0.04547835	0.78727224	147
SHBG	Tg	0.03048941	0.75208683	442
Tes	Tg	0.01942802	0.79720435	723
Tg	Tg	1	0	945
TIBC	Tg	0.17701201	1.58E-04	451
WBC	Tg	0.20228977	7.65E-09	801
Alb	TIBC	0.20943302	1.07E-04	452
ALT	TIBC	0.0172675	0.86364788	391
AST	TIBC	0.04371033	0.60220965	451
B12	TIBC	-0.1240229	0.04336036	453
BASOS	TIBC	-0.0475421	0.79078331	128
Ca	TIBC	0.24500136	2.57E-06	453
Chol	TIBC	0.27320845	1.02E-07	451
CK	TIBC	-0.0288315	0.76542847	446
Cor	TIBC	0.00140679	0.98778824	452
D	TIBC	-0.0873056	0.21340979	440
EOS	TIBC	0.01759907	0.92818058	128
FE	TIBC	0.04419042	0.59611788	455
Fer	TIBC	-0.197565	2.85E-04	455
Fol	TIBC	-0.0031307	0.97328236	452
FT	TIBC	0.0246102	0.84415241	288
GGT	TIBC	0.26938736	1.42E-07	454
Glu	TIBC	0.03565067	0.70251313	447
Hb	TIBC	0.2149454	7.07E-05	447
HCT	TIBC	0.28415968	0.0085536	128
HDL	TIBC	0.12983418	0.03326381	449
hsCRP	TIBC	0.0773785	0.28792432	450
K	TIBC	-0.0191783	0.85111048	449
LDL	TIBC	0.16104326	0.00555336	441
LYMPHS	TIBC	0.00728907	0.96715687	128
MCH	TIBC	-0.0401322	0.8361949	128
MCHC	TIBC	-0.07495	0.65208073	128
MCV	TIBC	0.05177289	0.77675523	128

Mg	TIBC	0.14568938	0.01255365	455
MONOS	TIBC	0.10223969	0.48665357	128
MPV	TIBC	0.16993883	0.18798223	126
Na	TIBC	-0.0806394	0.25798098	449
NEUT	TIBC	0.10592976	0.47290115	128
PLT	TIBC	0.17791567	0.16086825	127
RBC	TIBC	0.32454549	0.00177464	129
RDW	TIBC	0.01875409	0.92225612	128
SHBG	TIBC	0.02933348	0.76360462	437
Tes	TIBC	0.07506726	0.30932756	444
Tg	TIBC	0.17701201	0.00161857	451
TIBC	TIBC	1	0	455
WBC	TIBC	0.16876018	3.72E-04	441
Alb	WBC	0.10068484	0.12842274	449
ALT	WBC	0.08468949	0.17571086	533
AST	WBC	0.00335925	0.97008083	460
B12	WBC	0.01749697	0.81573963	790
BASOS	WBC	0.07517343	0.59497765	160
Ca	WBC	0.10340735	0.02267483	792
Chol	WBC	0.10090075	0.02640894	802
CK	WBC	-0.0438793	0.4576397	787
Cor	WBC	0.03727543	0.67927161	462
D	WBC	-0.0524961	0.34678373	788
EOS	WBC	0.21396975	0.03620274	160
FE	WBC	0.03078985	0.75208683	445
Fer	WBC	0.03010866	0.64381662	813
Fol	WBC	-0.0776383	0.12317352	774
FT	WBC	0.03662674	0.75743433	296
GGT	WBC	0.11616084	0.06100992	460
Glu	WBC	-0.0379772	0.53366309	798
Hb	WBC	0.16015098	6.45E-05	820
HCT	WBC	0.13822368	0.24201162	161
HDL	WBC	0.03306916	0.60106686	796
hsCRP	WBC	0.21982809	6.25E-06	528
K	WBC	-0.0456663	0.4295504	780
LDL	WBC	0.06100517	0.25798098	785
LYMPHS	WBC	0.33778242	1.68E-04	160
MCH	WBC	-0.1606951	0.15275687	161
MCHC	WBC	-0.0342993	0.83938504	161
MCV	WBC	-0.0696339	0.63483433	161
Mg	WBC	-0.038317	0.53442477	776
MONOS	WBC	0.42060522	6.83E-07	160
MPV	WBC	-0.1179906	0.34385462	160

Na	WBC	-0.0588366	0.28614935	782
NEUT	WBC	0.7233403	0	160
PLT	WBC	0.15913381	0.16007966	160
RBC	WBC	0.24738796	0.01094013	161
RDW	WBC	-0.0194606	0.90564582	161
SHBG	WBC	-0.023598	0.81183065	451
Tes	WBC	-0.0266277	0.72226809	728
Tg	WBC	0.20228977	1.92E-07	801
TIBC	WBC	0.16876018	0.00318521	441
WBC	WBC	1	0	833
